# Hyperactive Rac converts sublethal to lethal trogocytosis in vivo

**DOI:** 10.1073/pnas.2534451123

**Published:** 2026-04-06

**Authors:** Lauren Penfield, Abhinava K. Mishra, Morgan Smith, Denise J. Montell

**Affiliations:** ^a^Molecular, Cellular, and Developmental Biology Department, University of California, Santa Barbara, CA 93106; ^b^Department of Biology, University of Louisville, Louisville, KY 40292

**Keywords:** phagocytosis, trogocytosis, *Drosophila*, CAR-M, ovary

## Abstract

The small GTPase Rac is an essential regulator of cell shape, migration, macropinocytosis, and phagocytosis. We found that expression of constitutively active Rac^G12V^ is sufficient to cause a few migratory cells called border cells to cannibalize neighboring nurse cells in the *Drosophila* ovary. Building on that insight, we engineered mammalian Rac-enhanced chimeric-antigen-receptor macrophages (RaceCAR-Ms) to avidly engulf and kill cancer cells. Here, we investigate the cellular and molecular mechanisms by which border cells efficiently kill the much larger nurse cells. Surprisingly, wild type border cells normally nibble on nurse cells as they migrate between them, and Rac^G12V^ causes border cells to take larger, lethal bites. These larger bites trigger rapid germline shrinkage, nuclear damage, and caspase activation, which spreads through the nurse cell syncytium. Then, many somatic follicle cells join in to engulf the dying germline. Rac and the engulfment receptor Draper are critical for both sublethal and lethal nibbling (trogocytosis). Using clonal analysis, we show small groups of follicle cells expressing Rac^G12V^ induced caspase activation in neighboring follicle cells while larger Rac^G12V^ clones were required to cause germline killing. Increasing Draper expression or JNK activity in border cells also caused germline death, in a Rac-independent manner, suggesting that border cells can be activated to kill through multiple mechanisms. The series of events elucidated here reveals how hyperactivated Rac expressed in a few cells can trigger destruction of a much larger mass.

## Significance

Rac is a key protein in the cellular eating process called phagocytosis. Rac hyperactivity enhances the consumption of tumor cells by chimeric antigen receptor-macrophages (CAR-M), a promising type of cellular immunotherapy. Elucidating the mechanisms by which hyperactive Rac enhances cell killing may lead to improvements in CAR-M. Key insights into the in vivo effects of Rac have come from studying *Drosophila* oogenesis. Here, we report molecular and cellular mechanisms by which hyperactivated Rac stimulates migratory cells in the fly ovary to engulf and kill a group of cells ~50X larger, ultimately destroying the entire tissue. These insights have implications for how hyperactivating Rac might improve antibody and CAR-M therapies for cancer and other diseases.

The ability of one cell to eat another arose early in the evolution of eukaryotic cells and is a prevalent behavior in both unicellular and multicellular organisms (1–3). Many protists survive by engulfing smaller cells (4). In animals, macrophages and neutrophils are innate immune cells that specialize in eating and killing pathogens as well as stressed or diseased cells. Macrophage-mediated phagocytosis, which is defined as the engulfment of whole particles or cells >0.5 µm in diameter (Table 1), is also essential for tissue homeostasis; for example, human macrophages remove millions of senescent red blood cells every second. Macrophages also consume dying or dead cells to promote normal development and wound healing and to prevent inflammation. Macrophages consume neutrophils and T-cells to resolve inflammatory responses (5). They remove autoreactive B and T cells to avert autoimmune disease, and macrophage engulfment of incipient tumor cells contributes to cancer prevention. However, in some cases, cancer cells have co-opted this behavior. Tumor cells can feed on T cells, simultaneously suppressing antitumor immune attack and scavenging nutrients (6–9).

**Table 1.** Glossary of terms

Engulfment	A general term describing the process by which one cell internalizes another cell, particle, fragment, fluid, or object
Cellular cannibalism	One cell eating another, in whole or in part, alive or dead
Phagocytosis	The internalization of whole objects or cells >0.5 microns in diameter
Trogocytosis	Uptake of membrane fragments from one living cell into another during direct contact (also referred to as nibbling)
Lethal trogocytosis	Trogocytosis that causes target cell death
Sublethal trogocytosis	Trogocytosis that allows the target cell to live

Chimeric-antigen-receptor (CAR) macrophage therapy harnesses the ability of macrophages to engulf and kill harmful cells (10, 11). Peripheral blood monocytes are collected from cancer patients and engineered to express a CAR that recognizes cancer cells and triggers engulfment. Such CAR-macrophage or CAR-monocyte (CAR-M) immunotherapies are in clinical trials and are safe. However a key challenge is increasing the lethal phagocytic capacity of CAR-Ms (12). Some CAR-Ms take up pieces of cells rather than whole cells, in a nibbling process termed trogocytosis [(13) and Table 1]. Trogocytosis can either kill target cells or spare them. Converting sublethal to lethal trogocytosis or to whole-cell phagocytosis is a major goal for the cancer immunotherapy field (14). Therefore, identifying mechanisms that enhance engulfment of living target cells in native, 3D environments is essential. One way to learn more about how cells engulf and kill other living cells in vivo is to study such behaviors in relatively simple and accessible experimental organisms like the fruit fly *Drosophila* (15, 16).

Normal *Drosophila* oogenesis occurs in structures called egg chambers, which grow through 14 developmental stages as they mature. Egg chambers contain somatic follicle cells surrounding 16 germ cells, including 15 support cells called nurse cells and one egg cell (oocyte) (Fig. 1*A*). The germ cells are interconnected via cytoplasmic bridges called ring canals, through which the nurse cells donate cytoplasm to the oocyte. Afterward, nurse cells are no longer needed, and so-called stretch follicle cells kill and remove them (17) (Fig. 1*B*). Expression of engulfment genes in stretch follicle cells is required for nurse-cell death and consumption (18). The engulfment machinery includes transmembrane proteins such as integrins and the scavenger receptor Draper (Drpr, the fly homolog of human MEGF10/11/12), as well as intracellular signaling proteins like the kinases Src, Shark (Drosophila Syk), and Jun N-terminal kinase (JNK).

**Fig. 1.**
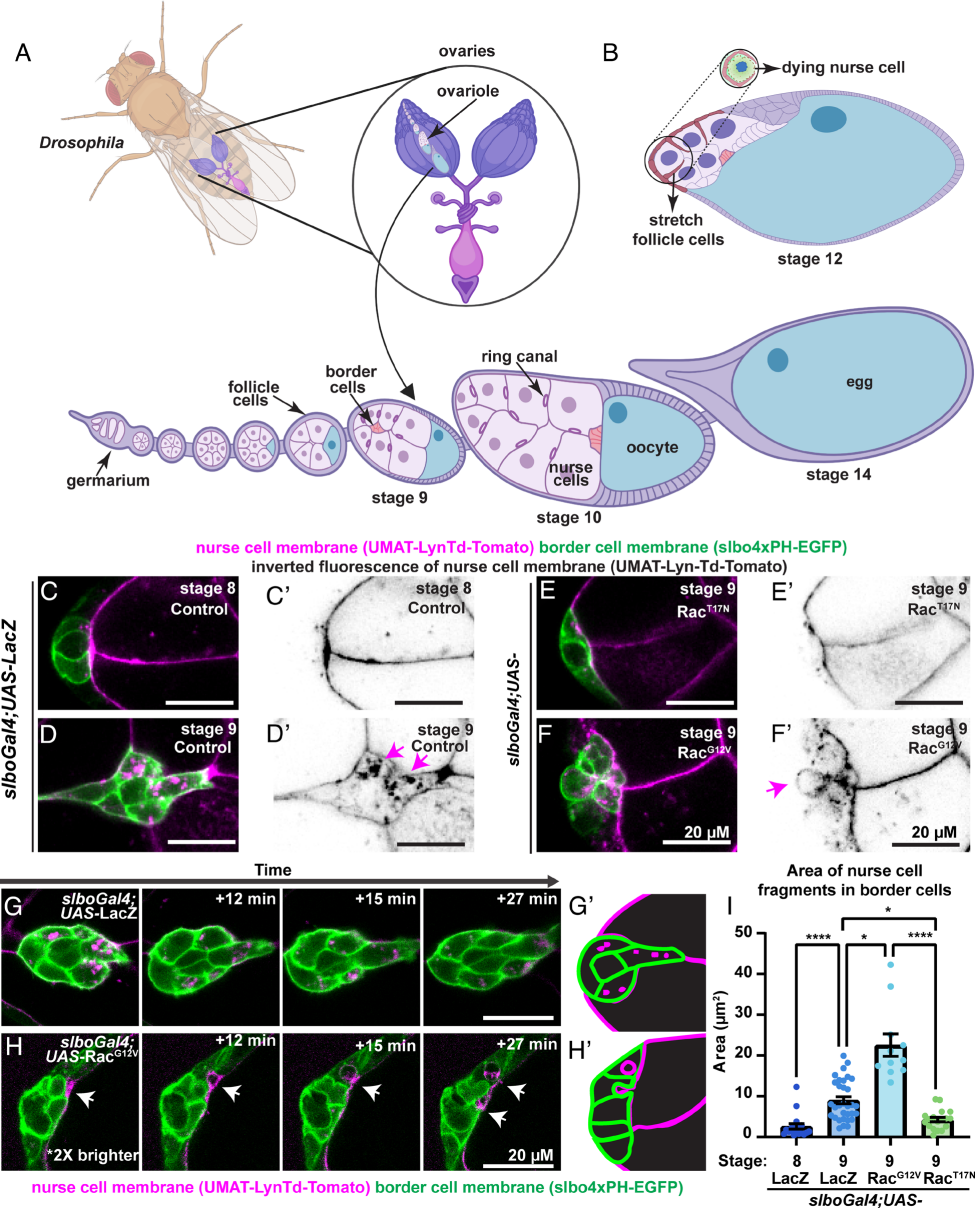
Wild type border cells nibble on nurse cells and Rac^G12V^ border cells take larger bites. (*A*) Schematic of Drosophila ovary and egg chamber development. (*B*) Stage 12 stretch cells surround and kill nurse cells. (*C* and *D*) Control stage 8 (*C*) and stage 9 (*D*) border cells expressing slbo4x-PH-EGFP (green); nurse cell membranes: UMAT-Lyn-TdTomato (magenta). (*C*’–*D*’) Inverted UMAT-Lyn-TdTomato. (*E* and *F*) Stage 9 border cells with slboGal4 driving UAS-Rac^T17N^ (*E*–*E*’) or UAS-Rac^G12V^ (*F*–*F*’). (*E*’–*F*’) Inverted fluorescence; magenta arrows: engulfed material. (*G* and *H*) Live imaging of slboGal4 driving UAS-LacZ (*G*) or UAS-Rac^G12V^ (*H*); brightness adjusted to match signal. White arrows: internalized UMAT-Lyn-TdTomato. See Movies S1 and S2. (*G*’–*H*’) Schematics of nurse cell membranes (magenta) in border cells (green). (*I*) Maximum nurse cell fragment area in border cells; mean ± SEM (n ≥ 10 egg chambers, ≥3 replicates). Kruskal–Wallis test; *P < 0.05, ****P < 0.0001. (Scale bar, 20 μm.)

In addition to this late-stage killing and removal of nurse cells, protein-deprived flies redirect nutrients from egg production to survival. This nutrient checkpoint occurs earlier, at stage 7/8 of egg development (19, 20). During nutrient stress, nurse cells undergo apoptosis, then all the surrounding follicle cells enlarge as they engulf the dying germline. Developmental nurse cell removal and starvation-induced egg chamber destruction employ some overlapping and some distinct mechanisms (17, 19, 21).

We recently discovered that expressing an activated form of the small GTPase Rac, which is generally required for trogocytosis and phagocytosis (22), in a few migratory follicle cells called border cells is sufficient to destroy the entire Drosophila egg chamber (15). Building on this insight, we showed that hyperactive Rac is also sufficient to cause mouse and human macrophages to engulf and kill chronically activated T-cells, likely contributing to the otherwise mysterious T cell loss that occurs in patients with dominant, activating Rac2 mutations. Moreover, this hyperengulfment behavior enhances CAR-M-mediated cancer cell phagocytosis and killing (15).

Here we report the cellular and molecular mechanisms by which Rac stimulates cell engulfment and killing of *Drosophila* egg chambers. We find that border cells normally nibble on their nurse cell substrate as they migrate. Germline nibbling requires the engulfment receptor Drpr and Rac. Border cells expressing hyperactive Rac (Rac^G12V^) take larger bites, triggering nurse cell shrinkage followed by loss of nuclear integrity, caspase activation, and apoptotic cell death, which spread rapidly through the nurse cell syncytium. Other follicle cells join in to consume the dying germline. Together these data show that Rac hyperactivity converts normal, sublethal nibbling of nurse cells to lethal trogocytosis and killing of a much larger cell mass, a major goal in CAR-M cancer immunotherapy.

## Results

### Wild Type Border Cells Nibble On Nurse Cells and Rac^G12V^ Border Cells Take Larger Bites.

The *Drosophila* ovary is composed of strands of developing egg chambers of increasing size and maturity (Fig. 1*A*). Germline and somatic stem cells reside in the germarium, at the anterior tip. Their progeny assemble into ovoid egg chambers composed of a syncytium of 16 interconnected germline cells surrounded by epithelial follicle cells. The most posterior germ cell differentiates into the oocyte; the rest become polyploid nurse cells. During development, the germline grows and the oocyte occupies an increasing percentage of the egg chamber. At developmental stage 8, anterior follicle cells termed polar cells induce 4 to 8 neighboring cells to become border cells. At stage 9, the border cells delaminate from the epithelium and migrate collectively (Fig. 1*A*), eventually reaching the anterior border of the oocyte by stage 10 (23). At stage 11, the nurse cells transfer the majority of their cytoplasm to the oocyte, which rapidly enlarges. At stage 12, anterior follicle cells termed stretch cells surround, isolate, kill, and engulf each nurse cell (Fig. 1*B*) (17).

The observation that Rac^G12V^ expression in a few border cells is sufficient to kill the entire egg chamber (15) raises the question as to how a handful of small cells can destroy a mass ~50-fold larger. Drpr contributes to Rac^G12V^-mediated killing (15), implying that Rac^G12V^-mediated killing requires the engulfment machinery. To visualize this process directly, we acquired high-magnification images of egg chambers in which nurse cell membranes were labeled with a membrane-targeting sequence from the Lyn tyrosine kinase fused to Tdtomato and expressed from a germline-specific enhancer/promoter (UMAT-LynTdTomato) (24). Border cell membranes were labeled with a plextrin homology (PH) domain from phospholipase C δ (PLCδ) fused to enhanced green fluorescent protein (EGFP) and expressed using an enhancer from the *slow border cells* (*slbo*) gene [*slbo*4x-PH-EGFP (25)]. We expressed *UAS-Rac^G12V^* or a control (*UAS-LacZ*) in border cells with *slboGal4* (25). While we expected Rac^G12V^-expressing border cells to engulf nurse cells, we were surprised to see UMAT-LynTdTomato fluorescence accumulating within control (*slboGal4;UAS-LacZ*) border cells. Whereas at stage 8, a few small puncta were visible within border cells (Fig. 1 *C* and *C’*), as border cells began migrating in stage 9, they accumulated more and larger nurse cell fragments (Fig. 1 *D* and *D’*). Thus, border cells normally nibble as they move between nurse cells.

Nibbling was dependent on Rac activity because dominant-negative Rac (Rac^T17N^) prevented the increase at stage 9 (Fig. 1 *E* and *E’*). The reduced nibbling by Rac^T17N^-expressing border cells was not a secondary consequence of their inability to migrate because Rac^G12V^-expressing border cells also fail to move, presumably due to an inability to polarize Rac activity (15, 26, 27), but accumulated even larger nurse cell fragments (Fig. 1 *F* and *F’*). Control border cells contained small puncta throughout their migration (Fig. 1 *G* and *G’* and Movie S1) whereas Rac^G12V^ border cells engulfed larger pieces of nurse cells (Fig. 1 *H* and *H’* and Movie S2). Thus, hyperactive Rac^G12V^ expression in border cells increases the sizes of engulfed fragments (Fig. 1*I*).

### The Engulfment Receptor Drpr Is Required for Border Cell Nibbling and Migration.

Since control border cells nibble on nurse cells, we investigated the expression and function of Drpr in border cells. Drpr expression increased in border cells compared to other follicle cells, specifically at stage 9 when nibbling increased and migration begins (Fig. 2 *A*–*D*). Then, we evaluated border cell nibbling and migration in *drpr* mutants, which are homozygous viable. In control egg chambers, 100% of border cell clusters complete migration by stage 10 (Fig. 2*E*) (28). In the *drpr* mutant, 60% of clusters failed to complete migration by stage 10 (Fig. 2 *F* and *G*) (29). Thus, Drpr is required for efficient border cell migration. Border cells lacking Drpr also engulfed significantly less nurse cell material compared to control border cells (Fig. 2 *H*–*J*). We conclude that border cells normally increase Drpr- and Rac-dependent nibbling in stage 9, as they migrate, without killing the germline.

**Fig. 2.**
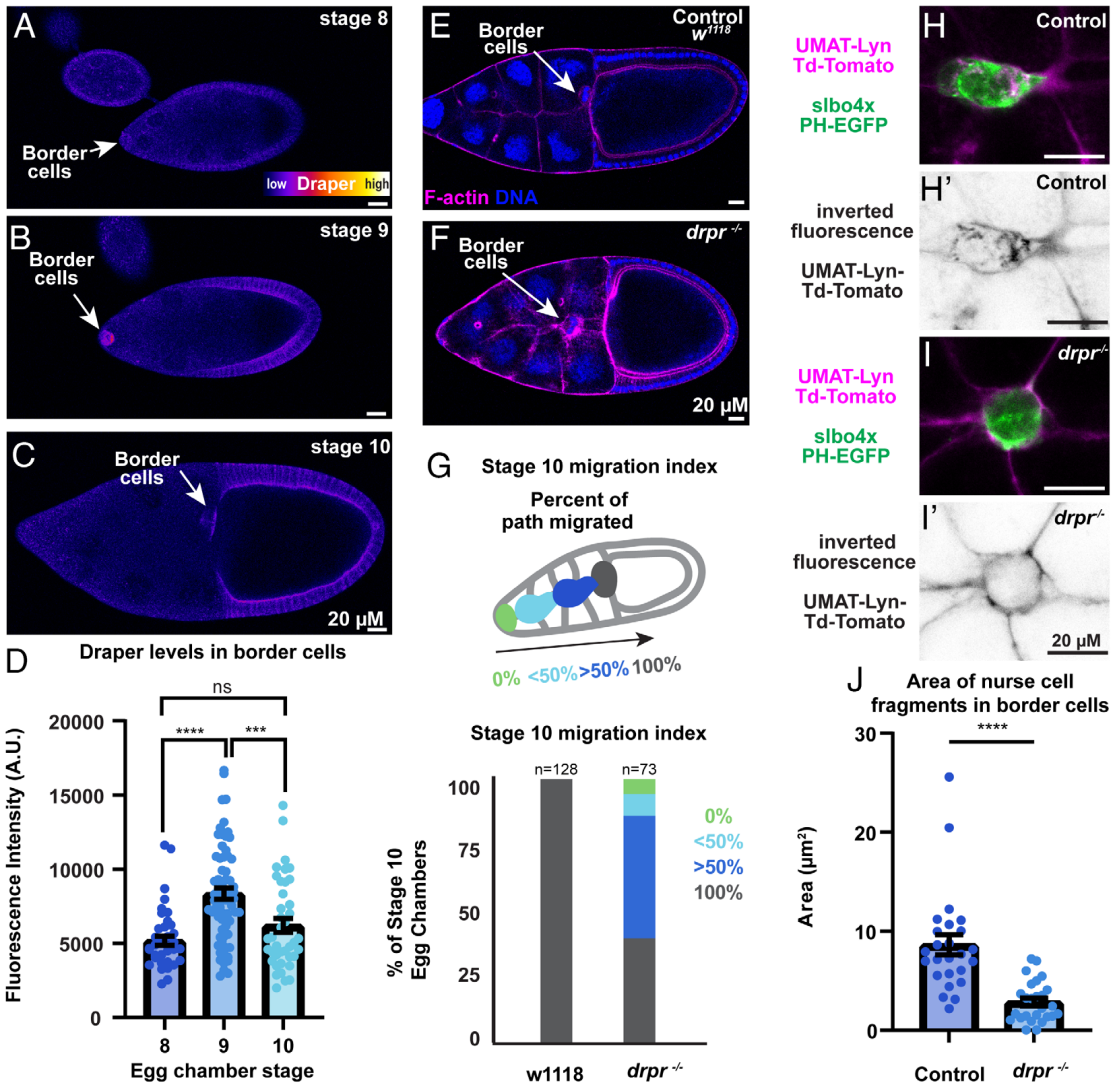
The engulfment receptor Drpr is required for border cell nibbling and migration. (*A*–*C*) Anti-Drpr staining in control egg chambers (c306Gal4;Gal80^ts^;UAS-LacZ); Fire LUT scale. (*D*) Drpr levels by stage in w^1118^ controls; mean ± SEM (n ≥ 40 border cell clusters, 3 replicates). ***P < 0.001, ****P < 0.0001. (*E* and *F*) Control and drpr^−/−^ egg chambers stained with phalloidin (magenta) and Hoechst (blue). (*G*) Border cell migration in control and *drpr* mutants (n = egg chambers; 8 to 9 replicates). (*H*–*I*’) Border cell membranes (slbo4X-PH-EGFP, green) and nurse cell membranes (UMAT-LynTd-Tom, magenta/black inverted) in control (*H*–*H*’) and drpr mutant (*I*–*I*’). (*J*) Area of largest nurse cell fragment in border cells; mean ± SEM (n ≥ 25 egg chambers, ≥3 replicates). ****P < 0.0001. (Scale bar, 20 μm.)

Together, these data suggest that the Rac^G12V^-induced effect on germ cells is an exaggeration of normal nibbling rather than a completely unnatural effect and indicate that increased Rac activity is sufficient to convert sublethal into lethal trogocytosis.

### Rac^G12V^ Border Cells Induce Cell Death That Spreads Through the Germline Syncytium.

To explore the sequence of events that result in germline killing, we tested if Rac^G12V^ nibbling preceded egg chamber death, we imaged egg chambers with border cells expressing *slbo*4x-PH-EGFP and nurse cells expressing ubiquitin-promoter-driven Histone fused to RFP (Ubi-His-RFP) in nuclei and UMAT-Lyn-TdTomato at plasma membranes (Fig. 3*A* and Movie S3). In control egg chambers, border cells migrate between nurse cells (Fig. 3 *A–A*’”), whereas Rac^G12V^-expressing border cells do not (15) (Fig. 3 *A*–*B*’’’ and *B*–*B*’’’). Control nurse cell nuclei maintain uniformly distributed His-RFP (Fig. 3*A* and Movie S3), but when border cells expressed Rac^G12V^, nurse cell nuclei underwent chromatin condensation (Fig. 3 *B’* and *B’’* and Movie S4) followed by fragmentation (Fig. 3*B’’’*). Higher magnification imaging showed that border cells normally extend protrusions between nurse cells but do not perturb the nuclei (Fig. 3 *C*–*C’’* and Movie S5). However, some Rac^G12V^ border cells extend protrusions that pinch both nurse cell membranes and nurse cell nuclei prior to chromatin condensation (Fig. 3 *D* and *E’’* and Movies S6 and S7). In some examples, chromatin condensation occurred simultaneously within the entire germline (Fig. 3 *B’–B’’’*, “synchronous”); however, we also observed examples where chromatin condensation clearly initiated in one cell that was interacting with a border cell and then spread to a neighboring nurse cell (Fig. 3 *E*–*E’’* and *F*). These data indicate that Rac^G12V^ border cells trigger germline death, which spreads rapidly through the syncytium.

**Fig. 3.**
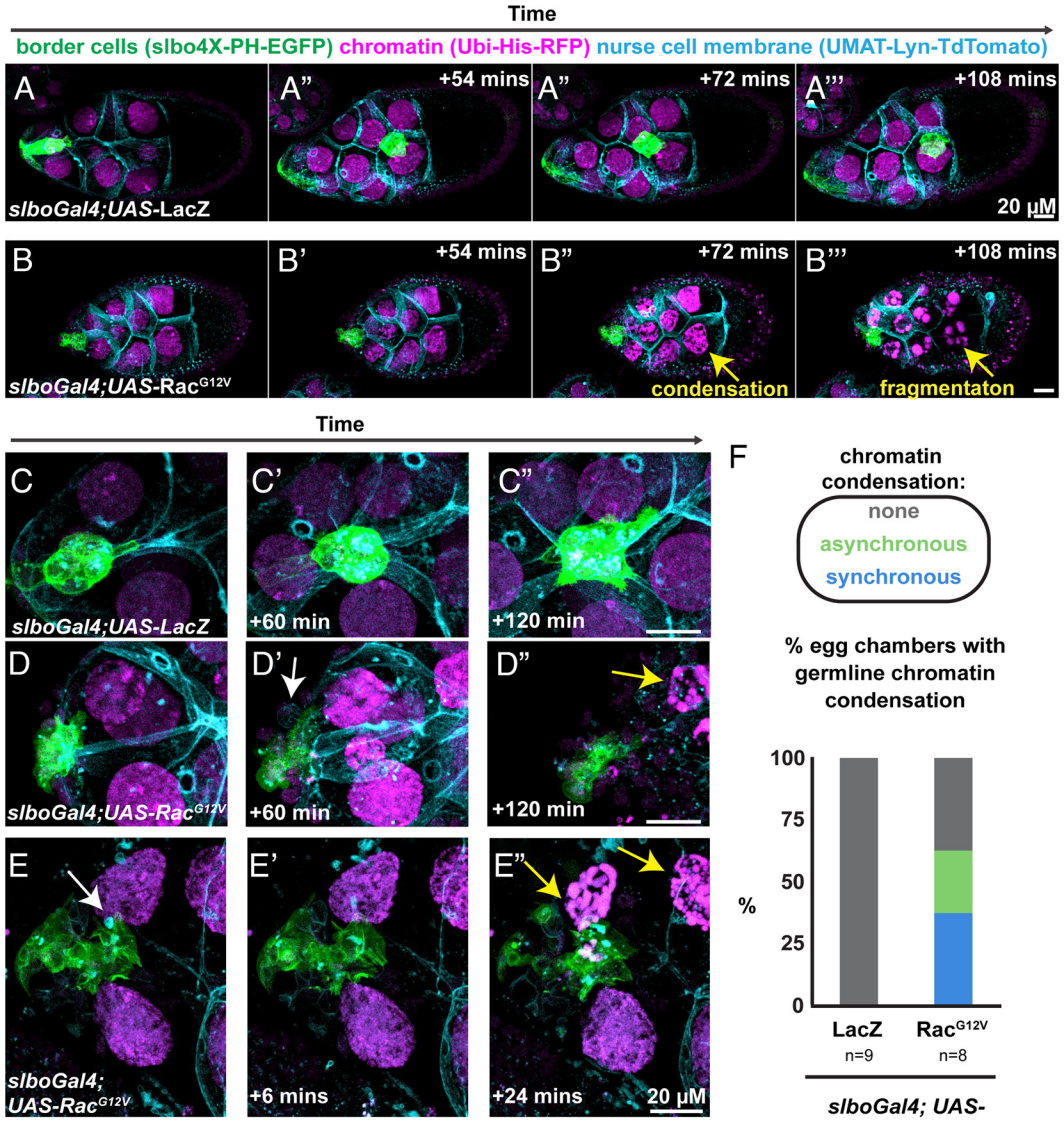
Rac^G12V^ border cells induce cell death that spreads through the germline syncytium. (*A* and *B*) Timelapse of control (slboGal4;UAS-LacZ) and slboGal4;UAS-Rac^G12V^ egg chambers. Border cells: slbo4xPH-EGFP (green); nuclei: UbiHisRFP (magenta); membranes: UMAT-Lyn-TdTomato (cyan). See Movies S3 and S4. (*C*–*E*) Higher magnification timelapse of control and Rac^G12V^-expressing border cells. White arrows: membrane pinching; yellow arrows: chromatin condensation/fragmentation. See Movies S5–S7. (Scale bar, 20 μm.) Lambda imaging with spectral unmixing; fluorescence intensity not comparable between movies. (*F*) Frequency of germline chromatin condensation (n ≥ 8 per condition). Synchronous: all nuclei condensed within 6 min; asynchronous: condensed >6 min apart.

### Rac^G12V^-Mediated Engulfment Triggers Nurse Cell Shrinking Prior to Caspase-Dependent Chromatin Condensation.

To investigate whether nurse cells undergo apoptotic cell death, we evaluated caspase activation by staining for the cleaved and activated form of Death caspase-1 (cDcp-1), an executioner caspase. Control egg chambers rarely if ever exhibited caspase activation or chromatin condensation (Fig. 4*A*). However, >50% of *slboGal4;UAS-Rac^G12V^* egg chambers exhibit condensed chromatin and >75% of those were positive for cDcp-1 (Fig. 4 *B*–*C*). Active caspases cleave nuclear lamins and other components of the nuclear envelope, resulting in loss of the nuclear permeability barrier (30, 31). Live imaging of control stage 9 egg chambers with a Ubi-GFP fused to a nuclear localization sequence (NLS) showed that control egg chambers retained the nuclear permeability barrier over time (Fig. 4 *D* and *D’* and Movie S8) whereas in egg chambers with border cells expressing Rac^G12V^ the nuclear permeability barrier was lost in a subset of germline cells and rapidly spread to neighboring cells (Fig. 4 *E* and *E’* and Movie S9). Together, these results suggest that Rac^G12V^ expressed in border cells triggers apoptosis in nurse cells.

**Fig. 4.**
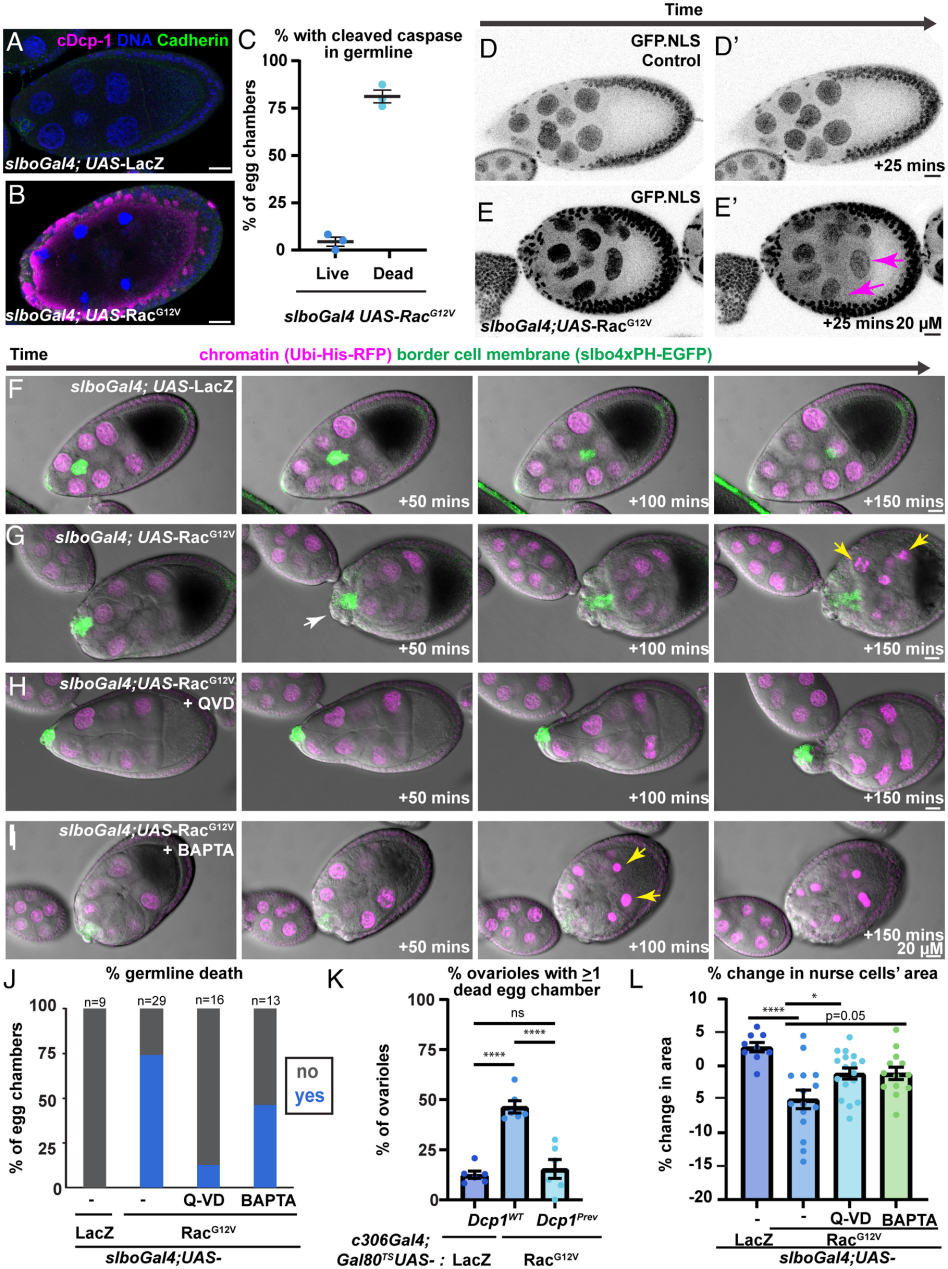
Rac^G12V^-mediated engulfment triggers nurse cell shrinking prior to caspase-dependent chromatin condensation. (*A* and *B*) cDcp-1 staining (magenta) in control (*A*) and slboGal4;UAS-Rac^G12V^ (*B*) egg chambers. (*C*) Percent cDcp-1+ egg chambers (≥15 per condition, 3 replicates). (*D*–*E*’) Timelapse of Ubi-NLS-GFP (inverted fluorescence) in control (*D*–*D*’) and slboGal4;UAS-Rac^G12V^ (*E*–*E*’). See Movies S8 and S9. (*F*–*I*) Timelapse of slboGal4;UbiHisRFP,slbo4XPH-EGFP with UAS-LacZ (*F*), UAS-Rac^G12V^ (*G*), UAS-Rac^G12V^+Q-VD-OPh caspase inhibitor (*H*), or UAS-Rac^G12V^+BAPTA-AM Ca^2+^ chelator (*I*). White arrows: germline shrinking; yellow arrows: chromatin condensation. (Scale bar, 20 μm.) See Movies S10–S13. (*J*) Percent egg chambers with chromatin condensation during live imaging (n = egg chambers; N = 2 to 4 replicates). (*K*) Percent ovarioles with chromatin condensation in fixed samples; mean ± SEM, each dot represents one experiment (≥14 egg chambers, ≥3 crosses). One-way ANOVA with Tukey. (*L*) Percent change in nurse cell area per 25 min; mean ± SEM (n ≥ 9 egg chambers, 2 to 4 replicates). One-way ANOVA with Tukey; *P < 0.05, ***P < 0.001.

We did not observe signs of lytic cell death such as nurse cell membrane rupture. 10 kDa dextrans accumulate in intercellular junctures between nurse cells in control egg chambers (*SI Appendix*, Fig. S1*A*) as previously reported (32). In *slboGal4;UAS-Rac^G12V^* egg chambers, 10 kDa dextrans still accumulate between nurse cells, and are excluded from the nurse cell cytoplasm despite chromatin condensation (*SI Appendix*, Fig. S1*B*). These data indicate that the large Rac^G12V^-mediated bites trigger apoptosis rather than necrosis within the nurse cell syncytium.

Cell shrinkage is a defining characteristic of apoptotic cells and distinguishes it from necrotic cell death, and apoptotic volume decrease can occur before caspase activation (33, 34). Egg chambers normally grow during stage 9 (Fig. 4*F* and Movie S10), so it was striking that *slboGal4;UAS-Rac^G12V^* egg chambers typically shrank after nurse cell membrane internalization and prior to chromatin condensation (Fig. 4*G* Movies S5–S7 and Movie S11). Consistent with the idea that nurse cells undergo apoptotic volume decrease prior to caspase activation, we also noticed an anteriorward flow of stretch follicle cells during shrinkage (Fig. 4*G* and Movie S11). Interestingly, stretch follicle cells start out as cuboidal and flatten out during stage 9, as a consequence of increasing germline volume (35), somewhat like a balloon stretching as it is inflated. Volume decrease then leads to unstretching, like a deflating balloon. While we considered the possibility that a Ca^2+^ influx that typically occurs upon plasma membrane damage might induce acto-myosin contractility in the nurse cells, we were unable to detect changes in Ca^2+^ influx (*SI Appendix*, Fig. S1*C*).

To further test possible contributions of caspase and/or calcium influx, we treated *slboGal4;UAS Rac^G12V^* egg chambers with a pancaspase inhibitor Q-VD-OPh (herein Q-VD), (Fig. 4*H*) or BAPTA-AM, a cell permeable calcium chelator (Fig. 4*I*). Within a 4-h imaging time frame, Q-VD significantly reduced the frequency of germline chromatin condensation despite some germline shrinkage, nuclear deformations, and changes in egg chamber shape (Fig. 4 *I* and *J* and Movie S12). We also observed a significant reduction in egg chamber death when we expressed Rac^G12V^ in border cells in homozygous loss-of-function caspase mutant *Dcp-1Prev* (Fig. 4*K*). However, as previously noted (36) *Dcp-1Prev* mutant egg chambers lack detectable follicle cells including border cells, making the rescue difficult to interpret (*SI Appendix*, Fig. S2). Interestingly, *slboGal4;UAS-Rac^G12V^* egg chambers underwent some morphological changes even when caspase activity was inhibited with Q-VD, so we assessed how germline shrinking was affected. Q-VD treatment significantly reduced the germline shrinking in *slboGal4;UAS-Rac^G12V^* egg chambers, yet it did not restore growth (Fig. 4*L*). We conclude that slboGal4;UAS-Rac^G12V^ induced nibbling and blocked growth and triggered caspase-dependent chromatin condensation and shrinkage.

Upon treatment with BAPTA-AM, Rac^G12V^-expressing border cells caused germline chromatin condensation in 46% of egg chambers examined by live imaging (Fig. 4*I* and Movie S13), compared to 79% of egg chambers killed without BAPTA-AM (Fig. 4*J*). Further, BAPTA-AM reduced the extent of germline shrinkage (Fig. 4 *I* and *L*). These data indicate intracellular calcium contributes to Rac-mediated germline death.

### Rac^G12V^-Induced Death Is Distinct From Both Starvation-Induced and Developmental Germline Death.

There are two situations in which follicle cells are known to engulf the germline (Table 2). There is a checkpoint at stage 7/8, and if females are protein-restricted, egg chambers do not develop further. Rather, the germline activates caspase, undergoes apoptosis and is engulfed by follicle cells, redirecting nutrients toward survival (19, 37). In protein-rich conditions, egg chamber development proceeds, nurse cells transfer their cytoplasm to the oocyte during stage 11, and as they shrink, the neighboring stretch follicle cells kill them, in a process that depends on the engulfment machinery (17).

**Table 2.** Features of germline death

Feature	Starvation	*Slbo*-Rac^G12V^	Development
Engulfment induces death	no^17,39^	yes^*^	yes^**^^29^
All follicle cells engulf	yes^17,39^	yes^*^	no^29^
Synchronous death	yes^17,39^	majority^*^	no^17-18^
Caspase accumulates	yes^17,39^	yes^*^	no^38^
Nurse cells acidified	no^37^	late^*^	yes^18^
Oocyte spared	no^17,39^	no^*^	yes^17,29^
Acute nuclear breakdown	yes^21^	majority^*^	no^21^
Draper required	yes^17,39^	yes^15^	yes^29^

^*^Data from this paper.

^**^Termed phagoptosis, engulfment genes are required.

In starvation mediated death, all follicle cells engulf the germline once it undergoes apoptosis while in developmental death, only stretch cells engulf nurse cells. We used UMAT-Lyn-TdTomato labeling to investigate follicle cell uptake of nurse cell material in control egg chambers compared to protein-starved and those with Rac^G12V^-expressing border cells. In control stage 8 egg chambers, some nurse cells had internal UMAT-LynTdTomato puncta, and many follicle cells contained small bits of UMAT-LynTdTom (Fig. 5 *A* and *A’* and *SI Appendix*, Fig. S3 *A* and *A’*). However, UMAT-LynTdTom puncta increased at stage 9 only within the border cells (Fig. 5 *B* and *B’* and *SI Appendix*, Fig. S3 *B* and *B’*). In starved egg chambers, main body follicle cells took up more germline material upon chromatin condensation (Fig. 5 *C* and *C’* and *SI Appendix*, Fig. S3 *C*–*D*’). In developmental death, the stretch cells engulfed the germline at stage 12 (Fig. 5 *D* and *D’*).

**Fig. 5.**
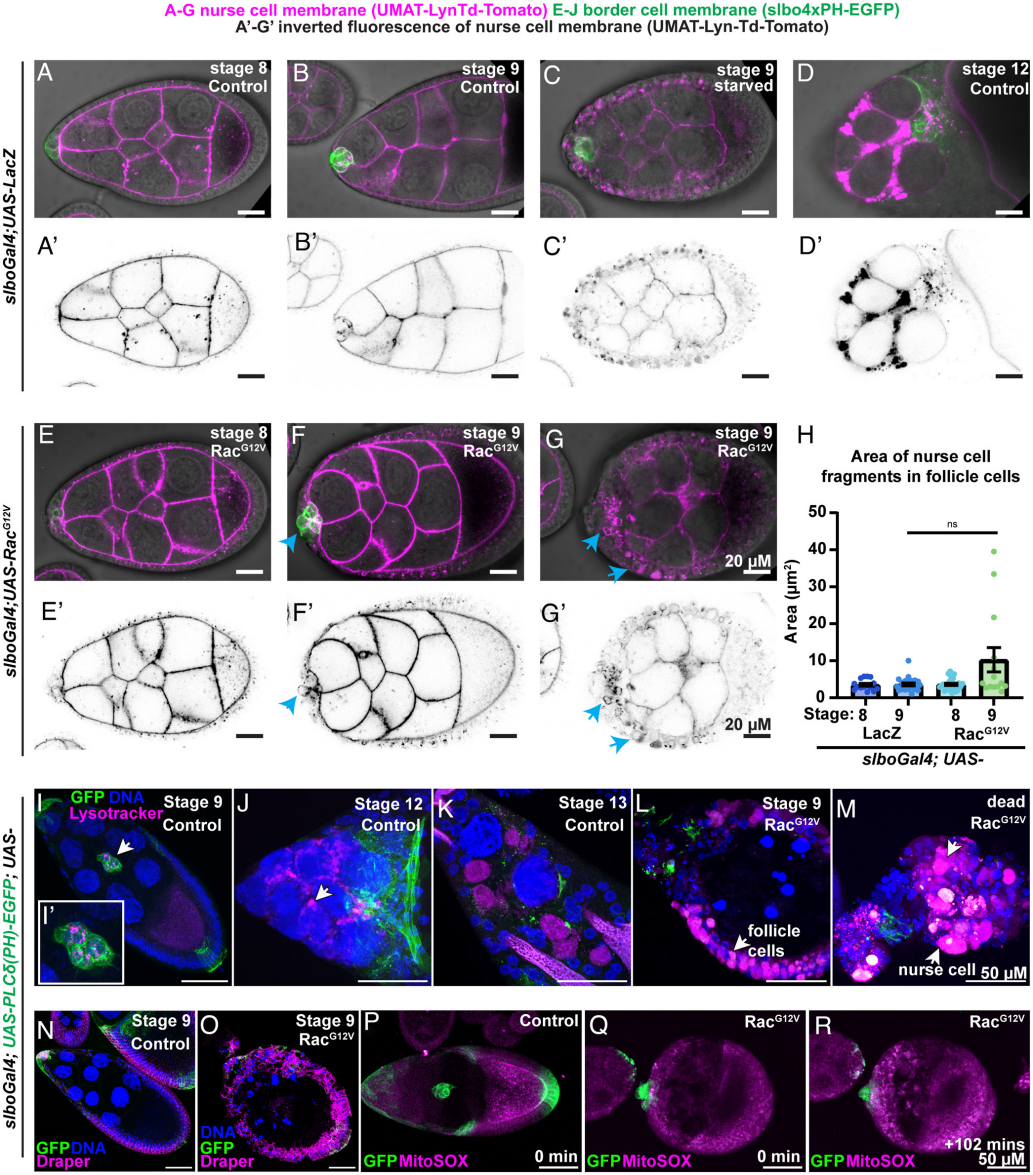
Rac^G12V^-induced death is distinct from both starvation-induced and developmental germline death. (*A*–*G*) DIC and fluorescence images showing border cell membranes (slbo4XPH-EGFP, green) and nurse cell membranes (UMAT-LynTdTomato, magenta). (*A*) control stage 8, (*B*) control stage 9, (*C*) starved stage 9, (*D*) control stage 12. (*E*–*G*) slboGal4;UAS-Rac^G12V^ at stage 8 (*E*), stage 9 (*F*), and upon death (*G*). (*A*’–*G*’) Inverted UMAT-LynTdTomato. (Scale bars, 20 μm.) (*H*) Nurse cell membrane fragment area in follicle cells; mean ± SEM, each dot represents one egg chamber (n > 10, ≥3 replicates). Kruskal–Wallis test; ns = not significant. (*I*–*M*) Lysotracker (magenta), GFP (green), DNA (blue) in control stage 9 (*I* and *I*’ magnified), stage 12 (*J*), stage 13 (*K*), and slboGal4;UAS-Rac^G12V^ stage 9 (*L* and *M*). (*N* and *O*) Anti-Drpr (magenta) and Hoechst (blue) in control (*N*) and slboGal4;UAS-Rac^G12V^;UAS-LifeActGFP (*O*). (*P*–*R*) Timelapse of slboGal4;UAS-PLCδGFP with UAS-LacZ (*P*) or UAS-Rac^G12V^ (*Q* and *R*) showing staining for mitochondrial superoxide using MitoSox dye (magenta in P-R). [Scale bars, 50 μm (*I*–*R*)]. See Movies S16 and S17.

In egg chambers expressing Rac^G12V^ in border cells, puncta in the majority of follicle cells remained small in stage 8 (Fig. 5 *E*–*E’*) and some intact stage 9 egg chambers (Fig. 5 *F*–*F’*). However, in shrunken and rounded stage 9 egg chambers, additional follicle cells increased their uptake of nurse cell membranes (Fig. 5 *G* and *H*), similar to starved egg chambers (Fig. 5*C*). We conclude that Rac^G12V^ border cells initiate germline death, which then triggers other follicle cells to join in to remove dying germ cells.

We also examined whether Rac^G12V^ shares features of developmental germline death, specifically germline acidification. During normal development, caspase activation does not initiate nurse cell death (38). Instead, stretch follicle cells surround and isolate individual nurse cells and secrete lysosomal enzymes that degrade each nurse cell asynchronously (18). To test if follicle cells acidify nurse cells during Rac-mediated death we used Lysotracker dye, which fluoresces upon acidification. In stage 9 controls, border cells exhibit larger lysotracker-positive particles than other follicle cells (Fig. 5 *I* and *I’* and *SI Appendix*, Fig. S3 *E*–*G’*), consistent with their nibbling behavior. At stages 12 to 13, abundant Lysotracker staining becomes evident first surrounding nurse cells (Fig. 5*J*), then within nurse cells (Fig. 5*K*). In *slboGal4;UAS-Rac^G12V^* egg chambers with germline chromatin condensation, most follicle cells had strong lysotracker staining (Fig. 5*L*), as they engulfed and degraded nurse cell material. Lysotracker accumulated in germline cells only late in the death process (Fig. 5*M*). We conclude that acidification is a late event in Rac^G12V^-induced germline death.

Live imaging revealed lysotracker-positive puncta in border cells (*SI Appendix*, Fig. S3 *E*–*F*’ and Movie S14) compared to faint lysotracker signal in other follicle cells (*SI Appendix*, Fig. S3 *G*–*G’*), which did not change substantially over time (*SI Appendix*, Fig. S3*E* and Movie S14). In *slboGal4;UAS-Rac^G12V^* egg chambers, lysotracker accumulated robustly in and around border cells (*SI Appendix*, Fig. S3 *H*-*I*’ and Movie S15) and in smaller puncta within distant follicle cells (*SI Appendix*, Fig. S3 *J*–*J’*). Lysotracker positive puncta in follicle cells became larger over time even prior to chromatin fragmentation (*SI Appendix*, Fig. S3*H*, +75 mins, and Movie S15). Nurse cells contained small lysotracker-positive puncta but there was no detectable change in lysotracker upon chromatin condensation (*SI Appendix*, Fig. S3*H*, +177 min and Movie S15). These data suggest that acidification of the nurse cells does not initiate germline death.

We next assessed Drpr levels. In starvation-mediated death, all follicle cells increase Drpr levels after germline nuclei begin to condense (39), while in developmental death, stretch cells specifically increase Drpr prior to nurse cell death (29). Drpr is normally expressed in all follicle cells (Fig. 5*N*), as previously reported (39). Drpr levels increased in all follicle cells in *slboGal4;UAS-Rac^G12V^* egg chambers with condensed germline chromatin (Fig. 5*O*), as they engulfed and consumed the dying cells.

Drpr overexpression in all follicle cells induces oxidative stress in anterior follicle cells (40), so we assessed the effect of border cell Rac^G12V^ expression on superoxide production using a mitochondrial superoxide sensor, MitoSOX. Compared to controls (Fig. 5*P* and Movie S16), MitoSOX increased in follicle cells and in the germline over time in *slboGal4;Rac^G12V^* egg chambers, after egg chambers shrank (Fig. 5 *Q* and *R* and Movie S17), concomitant with increased Lysotracker and Drpr labeling and consumption of the dead germline. We conclude that Rac-mediated death shares features of developmental and starvation-mediated death but is a distinct process as summarized in Table 2.

### Drpr-JNK Signaling Functions in a Parallel Death-Inducing Pathway.

JNK is critical for germline engulfment in response to starvation (39), developmental nurse cell death (29), and Drpr-induced germline death (40). We tested the role of JNK, if any, in Rac^G12V^-induced death, using *puc*-LacZ, a JNK activity reporter, and an antibody that recognizes the phosphorylated, active form (pJNK) in *slboGal4;UAS-Rac^G12V^* egg chambers. Border cells normally exhibit increased *puc-*LacZ staining (Fig. 6*A*) suggesting elevated JNK activity (41). In *slboGal4;UAS-Rac^G12V^* egg chambers, a few additional follicle cells show increased *puc*-lacZ (Fig. 6*B*), and more follicle cells gained JNK activity as the germline died (Fig. 6 *C* and *D*). Rac^G12V^ expression resulted in a 1.2-fold increase in pJNK levels compared to neighboring cells with endogenous Rac (Fig. 6 *E*–*H*).

**Fig. 6.**
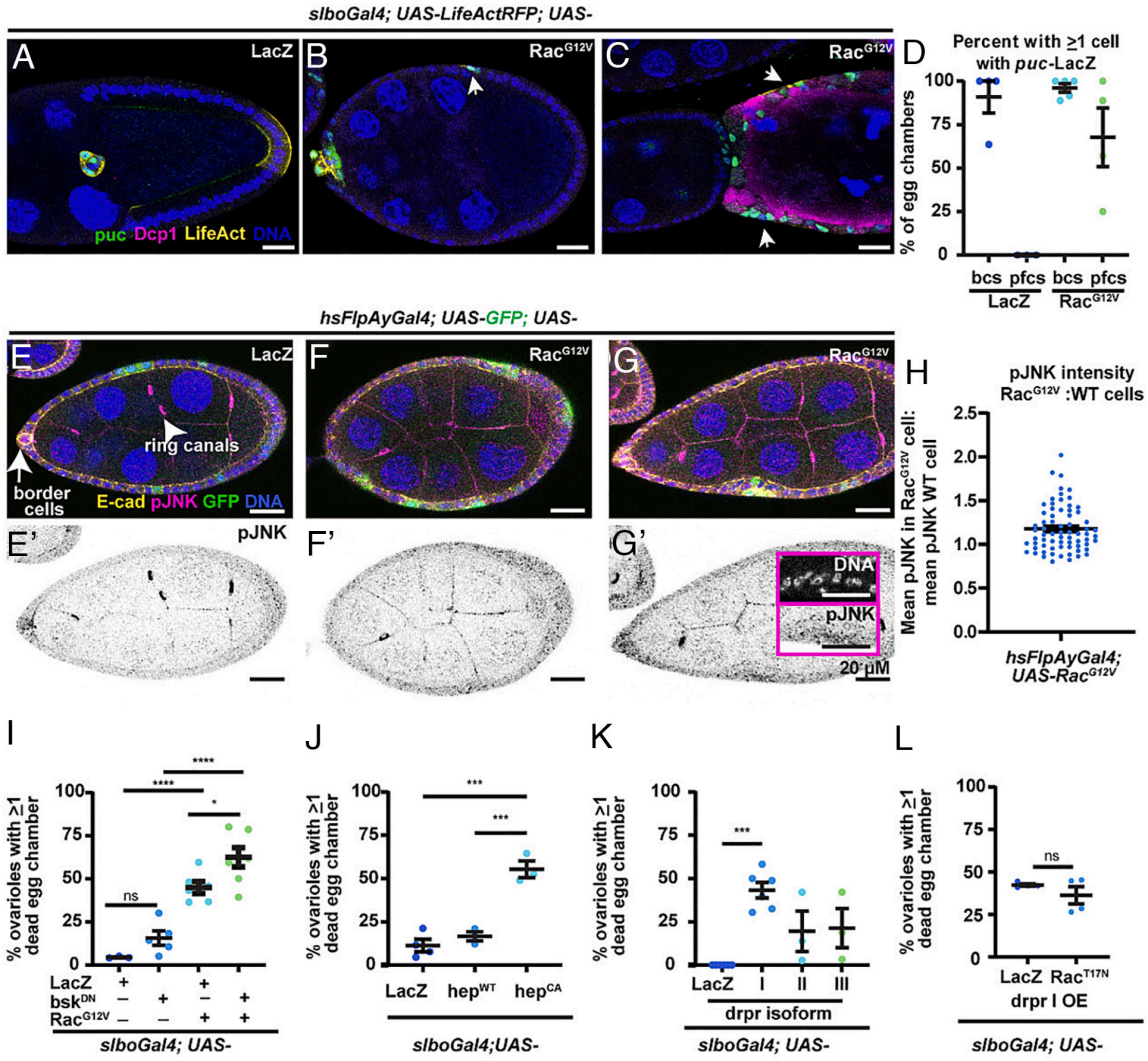
Drpr-JNK signaling functions in a parallel death-inducing pathway. (*A*–*C*) Control (slboGal4;UAS-LifeActRFP,puc-LacZ) (*A*) or slboGal4;UAS-Rac^G12V^,puc-LacZ (*B* and *C*) egg chambers. Arrowheads: puc-LacZ+ cells. (*D*) Percent puc-LacZ+ cells. Bc: border cells; pfc: posterior follicle cells. Each dot represents one replicate (≥9 egg chambers). (*E*–*G*’) hsFlpAyGal4 driving UAS-LacZ (*E*) or UAS-Rac^G12V^ (*F* and *G*) with UAS-GFP. pJNK shown in magenta; (*E*’–*G*’) inverted grayscale. (*H*) Fold change in pJNK (GFP+ vs GFP- cells); mean ± SEM from 3 replicates (≥10 clones each). Wilcoxon test, ****P < 0.0001. (*I*–*L*) Percent ovarioles with dying egg chambers; mean ± SEM. Each dot represents one replicate (≥18 egg chambers). UAS-LifeActRFP expressed in all lines. ANOVA with Tukey (*I*–*K*) or Mann–Whitney (*L*). ***P < 0.001, **P < 0.01, ns = not significant.

To test if JNK activity was required for border cells to induce death, we coexpressed dominant negative (DN) JNK (*UAS-Bsk^DN^*) or a control (*UAS-LacZ*) with *UAS-Rac^G12V^* using *slboGal4* or another border cell driver, *c306Gal4*. Bsk^DN^ did not reduce *Rac^G12V^-*induced germline death when expressed with *c306Gal4* (*SI Appendix*, Fig. S4*A*) and even increased when driven by *slboGal4* (Fig. 6*I*). We conclude that JNK activity is not required in border cells for Rac^G12V^ to induce germline death.

To test if excess JNK activity was sufficient to induce death when expressed in border cells, we expressed constitutively active (CA) JNK (hep^CA^) with *slboGal4* or *c306Gal4;Gal80^ts^*. Hep^CA^ but not wild type JNK (hep^WT^) induced significant germline death (Fig. 6*J* and *SI Appendix*, Fig. S4 *B*–*D*). Similarly, overexpression of Drpr isoform I, which contains the signaling immunoreceptor tyrosine-based activation motif (ITAM), was sufficient to induce a significant increase in egg chamber death in ovarioles (Fig. 6*K* and *SI Appendix*, Fig. S4*D*); however death occurred later in egg chamber development compared to hepCA- or Rac^G12V^-induced death. Upon Drpr I overexpression, the majority of egg chambers survived past stage 8, in contrast to hep^CA^- or Rac^G12V^- driven with *c306Gal4* (*SI Appendix*, Fig. S4*E*). To test if the Drpr-JNK mediated death required Rac activity, we coexpressed dominant negative Rac^T17N^, which blocks the activity of all three Drosophila Rac proteins (27), and Drpr with *slboGal4*. Rac^T17N^ did not significantly rescue egg chamber death induced by Drpr overexpression (Fig. 6*L*). These data indicate that Drpr and JNK function in a parallel pathway to Rac to induce germline death.

### Rac^G12V^ Expression in Nonborder Follicle Cells Also Causes Egg Chamber Death.

To assess the effects of *UAS-Rac1^G12V^* in nonborder follicle cells, we used *PG150-Gal4* to drive expression in stretch cells, the cells that normally engulf nurse cells late in development (17) (Fig. 7*A*). PG150-driven Rac^G12V^ expression caused a range of phenotypes (Fig. 7 *B*–*E*), which we categorized as normal (Fig. 7*B*, indistinguishable from controls), intermediate (Fig. 7*C*, evident nurse cell shrinkage), or severe (Fig. 7*D*, nurse cell chromatin condensation and fragmentation), similar to early and late stages of apoptotic death observed in *slboGal4;UAS-Rac^G12V^*. The majority of *PG150-Gal4;UAS-Rac^G12V^* egg chambers died prior to stage 10 (Fig. 7*F*). We conclude that Rac^G12V^ expression in stretch cells is sufficient to induce death through similar steps as observed in *slboGal4;UAS-Rac^G12V^*.

**Fig. 7.**
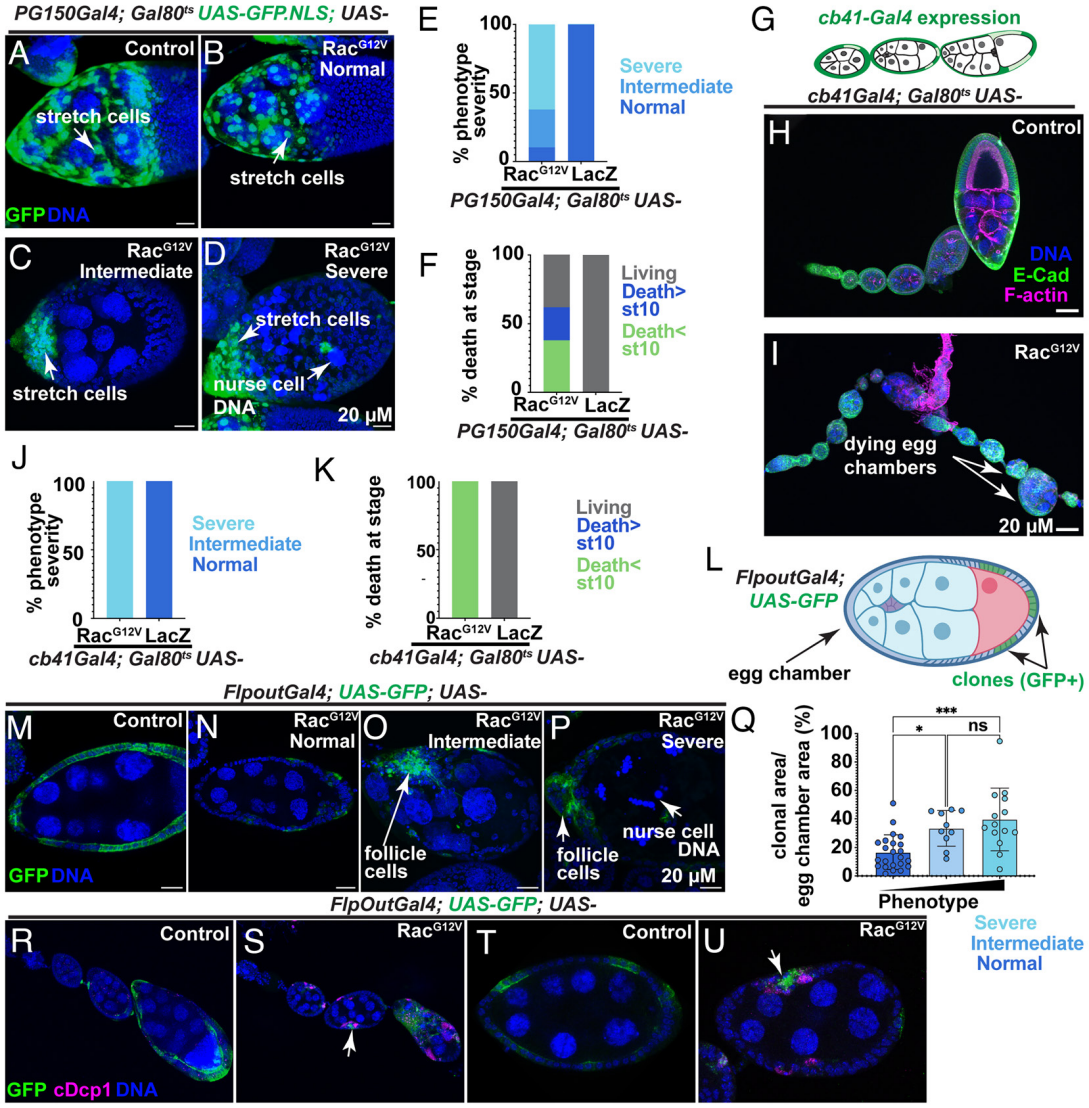
Rac^G12V^ expression in nonborder follicle cells also causes germline and follicle cell death. (*A*) Control stage 10 egg chamber (*PG150;Gal80^ts^,UAS-GFP.nls;UAS-LacZ*). (*B*–*D*) *PG150;Gal80^ts^;UAS-Rac^G12V^* egg chambers showing normal (*B*), intermediate (*C*), and severe (*D*) phenotypes. (*E* and *F*) Phenotype frequencies (*E*) and germline death (*F*) (N = 29). (*G*) Schematic of cb41-Gal4 expression. (*H* and *I*) Control or Rac^G12V^ expression with cb41-Gal4. (*J* and *K*) Phenotype severity and death stage (N = 22). (Scale bar, 20 μm.) (*L*) Schematic of FlpOut clonal expression. (*M*) *FlpOut-Gal4;UAS-mCD8-GFP* control. (*N*–*P*) *FlpOut-Gal4;UAS-mCD8-GFP;UAS-Rac^G12V^*. (*Q*) Death phenotype versus clonal size (N = 47). One-way ANOVA with Tukey’s test; *P < 0.05, ***P < 0.001. (*R*–*U*) *hsFlpAyGal4;UAS-GFP* controls (*R* and *T*) or with *UAS-Rac^G12V^* (*S* and *U*), stained for DNA (blue) and cDcp-1 (magenta). Arrows—dying cells adjacent to GFP+ cells.

We also expressed Rac^G12V^ with *cb41-Gal4*, which is expressed in a mosaic pattern that includes many follicle cells but not in border cells (Fig. 7*G*) (42). Expression of Rac^G12V^ with *cb41-Gal4* induced death even in young egg chambers, prior to the stage 7/8 checkpoint (Fig. 7 *H*–*K*). We conclude that other follicle cells are capable of killing the germline.

cb41 is expressed in most follicle cells while PG150 is expressed in ~50 cells (43), much more than the 4 to 8 border cells that express slboGal4, so it was not clear if a similarly small subset of nonborder cells expressing Rac^G12V^ would be capable of killing the germline. To investigate how many follicle cells expressing Rac^G12V^ were required to induce death, we used the FlpOut technique to drive Rac^G12V^ in clones of various sizes (Fig. 7 *L*–*P*). We found that egg chamber death was more likely to occur when >30% of follicle cells expressed Rac^G12V^, as measured by the fraction of egg chamber area containing Rac^G12V^-expressing follicle cells (Fig. 7*Q*). We conclude that Rac^G12V^-expressing follicle cells are sufficient to kill the germline but that a larger number of cells is required to match the potency of border cells. These results suggest that border cells are more efficient than other follicle cells at killing the germline.

Rac^G12V^-expressing cells induced caspase activation in neighboring follicle cells (Fig. 7 *R–U*). In addition to border cells, *slboGal4* is expressed in a small subset of posterior follicle cells at mid stage 9 (*SI Appendix*, Fig. S5*A*), and in *slboGal4; UAS-Rac^G12V^* egg chambers we observed death near slboGal4^+^ cells at the posterior (*SI Appendix*, Fig. S5 *B*–*B’’’*) and anterior, near border cells (*SI Appendix*, Fig. S5 *C*–*C’’’*). Thus, Rac^G12V^-induced death does not require germline-specific sensitivity to apoptosis or expression specifically in border cells.

## Discussion

Engulfment and killing of tumor cells by macrophages is a major mechanism by which CAR-macrophages and tumor-targeting antibodies such as rituximab and cetuximab exert antitumor activity (44–49). A significant challenge for these therapies is ensuring that macrophages achieve lethal clearance of sufficient tumor cells. Some FcγR^+^ myeloid cells instead perform sublethal trogocytic “nibbling,” which removes antigen–antibody complexes. Such “antigen shaving” promotes immune escape rather than cancer cell death (50–56). Therefore, elucidating the mechanisms that convert sublethal to lethal trogocytosis is of great interest. Sublethal nibbling and lethal whole cell engulfment are also frequent events in the normal development and homeostasis of organisms from amoebae to humans (55, 57). Therefore, these are conserved cellular behaviors.

Rac is an ancient and deeply conserved protein that serves as a critical node in the signaling and cytoskeletal pathways that govern chemotaxis and phagocytosis. Here, we report cellular and molecular mechanisms by which Rac hyperactivation in a handful of border cells in the fruit fly ovary is sufficient to convert sublethal to lethal trogocytosis, thereby eliminating a much larger mass of cells. We delineate the steps by which border cells kill germline cells (Fig. 8): Rac hyperactivity drives biting off of fragments about twice as large as normal, which triggers shrinkage in target cells followed by loss of the nuclear permeability barrier, caspase activation, and chromatin condensation and fragmentation. Ultimately all the follicle cells join in to consume the dying germline.

**Fig. 8.**
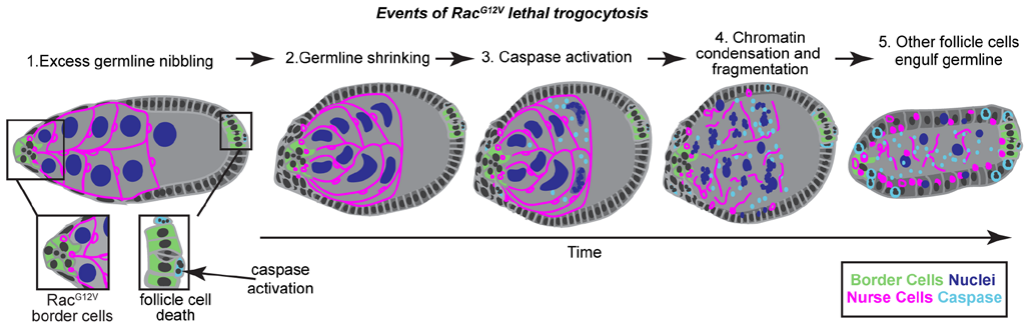
Schematic of steps in Rac^G12V^-mediated death.

We were surprised to find that border cells normally nibble on nurse cells as they migrate, in a Drpr- and Rac-dependent manner, raising the question as to the purpose of this sublethal trogocytosis. Nibbling may contribute to border cell motility, as Drpr expression increases as border cells initiate migration, and Drpr is required for border cells to complete their migration. Another possibility is that nibbling clears space between nurse cells as it has been shown that even tiny spaces much smaller than a single border cell reduce the energy barrier for their movement (32). Alternatively, border cells may consume nurse cell material for fuel as has been described for cannibalistic cancer cells (6). Alternatively or in addition, trogocytosis may allow border cells to sense germline health. In zebrafish for example, macrophages nibble on hematopoietic stem and progenitor cells to assess their health, and if the phagocytes sense oxidative stress they phagocytose and remove the stressed progenitor (58). No defects in nurse cell or oocyte development are detectable when border cell migration is inhibited, suggesting that nibbling does not contribute to other aspects of oogenesis. However, it is possible that under stress conditions, additional consequences of impairing border cell migration or nurse cell nibbling would become evident. In *C. elegans* development, endodermal cells engulf large, mitochondria-rich lobes from primordial germ cells, presumably to prune unfit organelles (59). Similar cell–cell interactions may be more widespread than previously appreciated, serving context-dependent functions. As in border cell nibbling, Rac1 is a central molecular player in sublethal and lethal trogocytosis as well as whole cell engulfment in most if not all of these examples (57).

The work presented here shows that there are multiple, related yet distinct mechanisms by which follicle cells can engulf and/or kill the germline. We find that Rac-mediated germline killing shares similarities to both starvation mediated germline engulfment and developmental engulfment of nurse cells and yet is distinct from both. Rac-mediated death is initiated by border cell trogocytosis of nurse cells, whereas starvation-induced death starts with germline apoptosis, and developmental death begins when stretch follicle cells enwrap and acidify individual nurse cells.

Our data indicate that constitutively activated Rac engages multiple pathways to promote germline killing. Rac is required for the formation of F-actin–rich protrusions that generate the phagocytic cup, and Rac hyperactivity enables border cells to take larger bites. We previously reported that *drpr* mutants partially rescue Rac-mediated germline death (15). The rescue appeared to prevent even the initiation of death, so it is likely that Drpr expression in border cells enhances their ability to take the larger bites required to convert sublethal to lethal trogocytosis. Prior work shows that Drpr promotes germline death by inducing oxidative stress (39) and driving acidification-mediated nurse-cell degradation during development (29). Rac is also known to activate NADPH oxidase to generate the oxidative burst in phagocytes, so Rac and Drpr may synergize to generate oxidative stress in addition to physical damage. We only observed acidification and oxidative stress in the germline late in the death process, after upregulation of Drpr expression in all follicle cells. Thus, only after nurse cells die, do Rac^G12V^-expressing egg chambers engage processes similar to those that are normally employed during stage 12.

Rac-induced death resembles starvation-induced death in that it depends on caspase-mediated apoptosis, the oocyte is not spared, and all follicle cells eventually engulf the dying germline. These similarities might arise from the egg chamber stage since Rac1^G12V^-expressing border cells kill the germline in stages 8 to 9, which is similar to starvation-induced death at stages 7 to 8. However unlike starvation-mediated death but like developmental death, follicle cells initiate Rac^G12V^-induced killing nonautonomously in a process that requires engulfment genes. In starvation-induced death, caspase-dependent germline apoptosis precedes engulfment. Intriguingly, treatment of *Drosophila* with rapamycin, which inhibits the nutrient sensor Target of Rapamycin (TOR), induces follicle cells to engulf the germline (60), and this effect is conserved in mammalian oogenesis. Thus, follicle cells can sense nutrient stress, either autonomously or nonautonomously, and may be primed to kill and engulf the germline under suboptimal conditions. However, the observation that Rac^G12V^ induces caspase activation in neighboring follicle cells indicates that Rac^G12V^-induced death does not require a germline-specific sensitivity to apoptosis. It is interesting that Rac^G12V^-expressing border cells engulf but do not kill polar cells (15), even when they are stretched and split, including obvious nuclear deformation, suggesting that polar cells are especially resistant to apoptosis. Identifying the tipping points, such as levels of Rac activity in the engulfing cell and vulnerabilities in the targets, that make the difference between sublethal and lethal engulfment behavior enhance both our understanding of homeostatic checkpoints and the ability to engineer therapeutic phagocytes to remove unwanted cells.

## Materials and Methods

Fly husbandry, ovary dissections, and immunostaining were carried out according to standard procedures. Detailed methods are described in the supplementary information. Live imaging was carried out as described (61).

### Image Analysis.

Death was scored per ovariole when germline chromatin became condensed and/or fragmented. Chromatin condensation was defined as histone signal enrichment in spots within nuclear boundaries; fragmentation as condensates splitting apart. Nurse cell area inside border cells was measured in ImageJ by drawing ROIs around membrane fragments. Germline area was traced from DIC images every 25 min until death or loss of focus. Migration was scored in stage 10 egg chambers as 0%, <50%, >50%, or 100% complete.

For fluorescence intensity, ROIs were drawn around cells of interest with background subtraction from regions outside the egg chamber. cDcp-1 and puc-LacZ positivity were scored manually based on signal enrichment.

### Statistical Analysis.

Inclusion criteria: undamaged samples, correct stage, in-focus images.

## Supplementary Material

Appendix 01 (PDF)

Movie S1. Control border cells accumulate nurse cell membrane puncta. *slboGal4*; *UAS-LacZ*; *slbo*4XPHEGFP(green, border cell membranes) and UMAT-LynTdTomato (nurse cell membranes, magenta). Single z-slices acquired every 3 minutes. Scale bar: 20 μm.

Movie S2. Rac^G12V^-expressing border cells take in larger nurse cell fragments. *slboGal4*; UAS-Rac^G12V^; *slbo4XPHEGFP* and UMAT-LynTdTomato. Single z-slices acquired every 3 minutes. Scale bar: 20 μm.

Movie S3. Control border cell migration. Egg chambers expressing *slboGal4*; *UAS-LacZ*, *slbo4*XPH-EGFP (green), UMAT-LynTd-Tomato (Cyan), and Ubi-Histone-RFP (magenta). Max z projections of images acquired every 6 minutes. Scale bar: 10 μm.

Movie S4. Germline death in *slboGal4*; UAS-Rac^G12V^. Egg chambers also expressed *slbo4*XPH-EGFP (green), UMAT-LynTd-Tomato (Cyan), and Ubi-Histone-RFP (magenta). Max z projections of images acquired every 6 minutes. Scale bar: 10 μm.

Movie S5. High magnification of control border cell migration in egg chambers from *slboGal4*; *UAS-LacZ*, *slbo*4XPH-EGFP (green), UMAT-LynTd-Tomato (Cyan), and Ubi-Histone-RFP flies. Max z projections of images acquired every 6 minutes. Scale bar: 20 μm.

Movie S6. High magnification of *slboGal4*; UAS-Rac^G12V^,. *slbo*4XPH-EGFP (green), UMAT-LynTd-Tomato (Cyan), and Ubi-Histone-RFP (magena), Max z projections of images acquired every 6 minutes. Scale bar: 20 μm.

Movie S7. Second higher magnification example of *slboGal4*; *UAS-Rac*^G12V^, *slbo*4XPH-EGFP (green), UMAT-LynTd-Tomato (Cyan), and Ubi-Histone-RFP (magena). Max z projections of images acquired every 6 minutes. Scale bar: 20 μm.

Movie S8. Control egg chambers retain the nuclear permeability barrier during stage 9. Egg chamber expressing Ubi-GFP.NLS (inverted grayscale fluorescence). Max z projections of images acquired every 5 minutes. Scale bar: 20 μm.

Movie S9. Posterior germline cell nuclei lose the permeability barrier, and the loss spreads through the syncytium during death. Egg chamber expressing slboGal4; *UAS-Rac*^G12V^ *slbo*4XPH-EGFP Ubi-GFP.NLS (inverted grayscale fluorescence). Max z projections of images acquired every 5 minutes. Scale bar: 20 μm.

Movie S10. Egg chamber growth during stage 9. Egg chambers expressing, *slboGal4*; *UAS-LacZ*, *slbo*4XPH-EGFP (green), UbiHisRFP (magenta). The differential interference contrast (DIC) image is overlaid with fluorescent channels. Single z-slices of images acquired every 5 minutes. Scale bar: 20 μm.

Movie S11. Egg chamber shrinkage prior to Rac^G12-^ induced chromatin condensation. Time-lapse series from egg chambers expressing slboGal4; *UAS-Rac*^G12V^ *slbo*4XPH-EGFP (green), UbiHisRFP (magenta). The differential interference contrast (DIC) image is overlaid with fluorescent channels.Single z-slices of images acquired every 5 minutes. Scale bar: 20 μm.

Movie S12. QVD rescues Rac^G12V^ mediated chromatin condensation. Time-lapse series from egg chambers expressing *slboGal4*; *UAS-Rac*^G12V^ *slbo4*XPH-EGFP (green), UbiHisRFP (magenta) and treated with 100 micromolar QVD for 30 minutes prior to imaging. The differential interference contrast (DIC) image is overlaid with fluorescent channels. Single z-slices of images acquired every 5 minutes. Scale bar: 20 μm.

Movie S13. BAPTA-AM treatment of *slboGal4*; *UAS-Rac*^G12V^ egg chambers. Time-lapse series from egg chambers expressing *slboGal4*; *UAS-Rac*^G12V^ *slbo*4XPH-EGFP (green), UbiHisRFP (magenta) treated with 50 micromolar BAPTA-AM. The differential interference contrast (DIC) image is overlaid with fluorescent channels. Single z-slices of images acquired every 5 minutes. Scale bar: 20 μm.

Movie S14. Lysotracker-positive puncta during normal border cell migration. Timelapse series of a (*slboGal4*;*UAS-LacZ*) expressing UbHisRFP (blue), *slbo*4PHEGFP (green) and stained for lysotracker-deep red (magenta). Max z-projection of images acquired every 3 minutes. Scale bar: 20 μm.

Movie S15. Lysotracker accumulation in border and follicle cells during Rac-induced death. Time-lapse series egg chambers expressing *slboGal4*;*UAS-Rac*^G12V^, UbHisRFP (blue), and *slbo4*PHEGFP (green) that are stained for lysotracker-deep red (magenta). max z-projection of images acquired every 3 minutes. Scale bar: 20 μm.

Movie S16. MitoSox staining (magenta) in control egg chambers expressing *slboGal4*; *UAS-LacZ*;*UASPH*(PLCδ)-*GFP* (green). Images acquired every 3 minutes.

Movie S17. MitoSox staining (magenta) in *slboGal4*;UAS-;Rac^G12V^;*UAS-PH(PLCδ)*-*GFP* (green) egg chambers. Images acquired every 3 minutes.

## Data Availability

All study data are included in the article and/or supporting information.
