# Supplementary material for: Hyperactive Rac converts sublethal to lethal trogocytosis in vivo
Source: Proc Natl Acad Sci U S A. 2026 Apr 6;123(15):e2534451123. doi: 10.1073/pnas.2534451123 (PMC13079925; doi:10.1073/pnas.2534451123)
Supplement: Supplementary file 1 [file pnas.2534451123.sapp.pdf]

Supplemental Table 1. Drosophila Fly Stocks

Stock	**Source**
w^{1118} *;slboGal4/CyO; UMAT-Lyn-TdTomato, slbo4XPH-EGFP/ (TM6B)*	Montell Lab stock, UMAT-Lyn-TdTomato (https://flybase.org/reports/FBtp0160063.html) and *slbo*4XPH-EGFP stocks gifts from Hsin-Ho Sung
UAS-Rac^{G12V}	Bloomington Drosophila Stock Center 6291
w^{1118}*; Sp/CyO;UAS-Rac*^{T17N}	D. Montell Lab stock
UAS-LacZ	Bloomington Drosophila Stock Center 3956
w^{1118}*; Sco/CyO; MKRS/TM6B*	D. Montell Lab stock
w^{1118}*; Sp/CyO; Drpr*$^{\Delta 5}$*/TM3*	Marc R. Freeman
w^{1118}*; slboGal4/CyO; UMAT-Lyn-TdTomato, UbiHisRFP; slbo4XPH-EGFP/TM6B*	Montell Lab stock (Dai & Guo et al., 2020)
Ubi-GFP(S65T)nls}2L P{ry[+t7.2]=neoFRT}40°/CyO	Bloomington Drosophila Stock Center 5629
c306Gal4; Gal80^{TS}	D. Montell Lab stock
yw;slboGal4/CyO; UAS-PLCδ(PH)-EGFP	D. Montell Lab stock<br>PLC-δ-EGFP is from BDSC 39693
slboGal4/CyO ; UASt-lifeAct-RFP/(TM6B)	D. Montell Lab stock
Dcp-1^{Prev}	Bloomington Drosophila Stock Center 63814
pucE69/TM6B (puc-LacZ)	Bergmann lab
UAS-Bsk^{DN}	Bloomington Drosophila Stock Center 6409
UAS-hep^{WT}	Bloomington Drosophila Stock Center 9308
UAS-hep^{CA}	Bloomington Drosophila Stock Center 9306
UAS-drpr I	Bloomington Drosophila Stock Center 67035
UAS-drpr II	Bloomington Drosophila Stock Center 67036
UAS-drpr III	Bloomington Drosophila Stock Center 67037
UAS-Src^{CA}	Bloomington Drosophila Stock Center 6410
hsFlpAyGal4; UAS-GFP	Bloomington Drosophila Stock Center 1929 and 4411
cb41Gal4; tubGal80^{TS}	Joceyln McDonald
P*G150Gal4; tubGal80*^{TS}*,UAS-GFP.nls/CyO*	Celeste Berg

Supplemental Figures

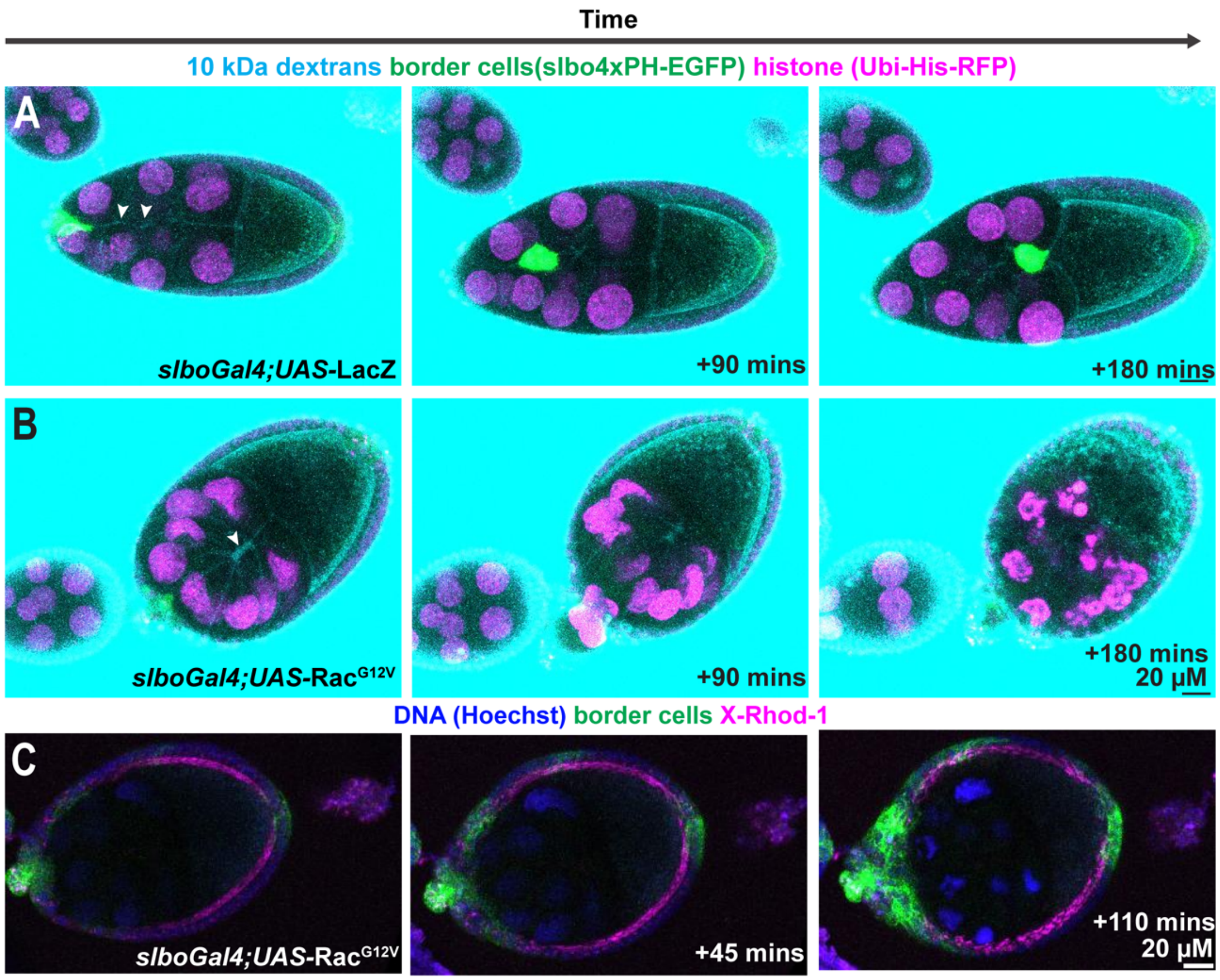

Figure S1. Germline cells do not have detectable breaches in the plasma membrane prior to chromatin condensation. (A-B) Images from a timelapse series of egg chambers overexpressing *UAS-LacZ* control(A) or *UAS-Rac*^{G12V}(B) with *slboGal4*. Border cells are marked with *slbo*4X-PH-EGFP (green). Nuclei are labeled with UbiHis-RFP (magenta). Egg chambers are incubated with 10 kDa dextrans conjugated with Alexa 647 (cyan). Arrowheads point to dextrans that accumulate between the nurse cells. (C) Images from a timelapse series of an egg chamber expressing *slboGal4;UAS-Rac*^{G12V} *;UAS-PH(PLCδ)-EGFP* (green) and stained with XRhod-1, a calcium indicator (magenta), and Hoechst to label DNA (blue). Scale bars: 20 μm.

Figure S2.

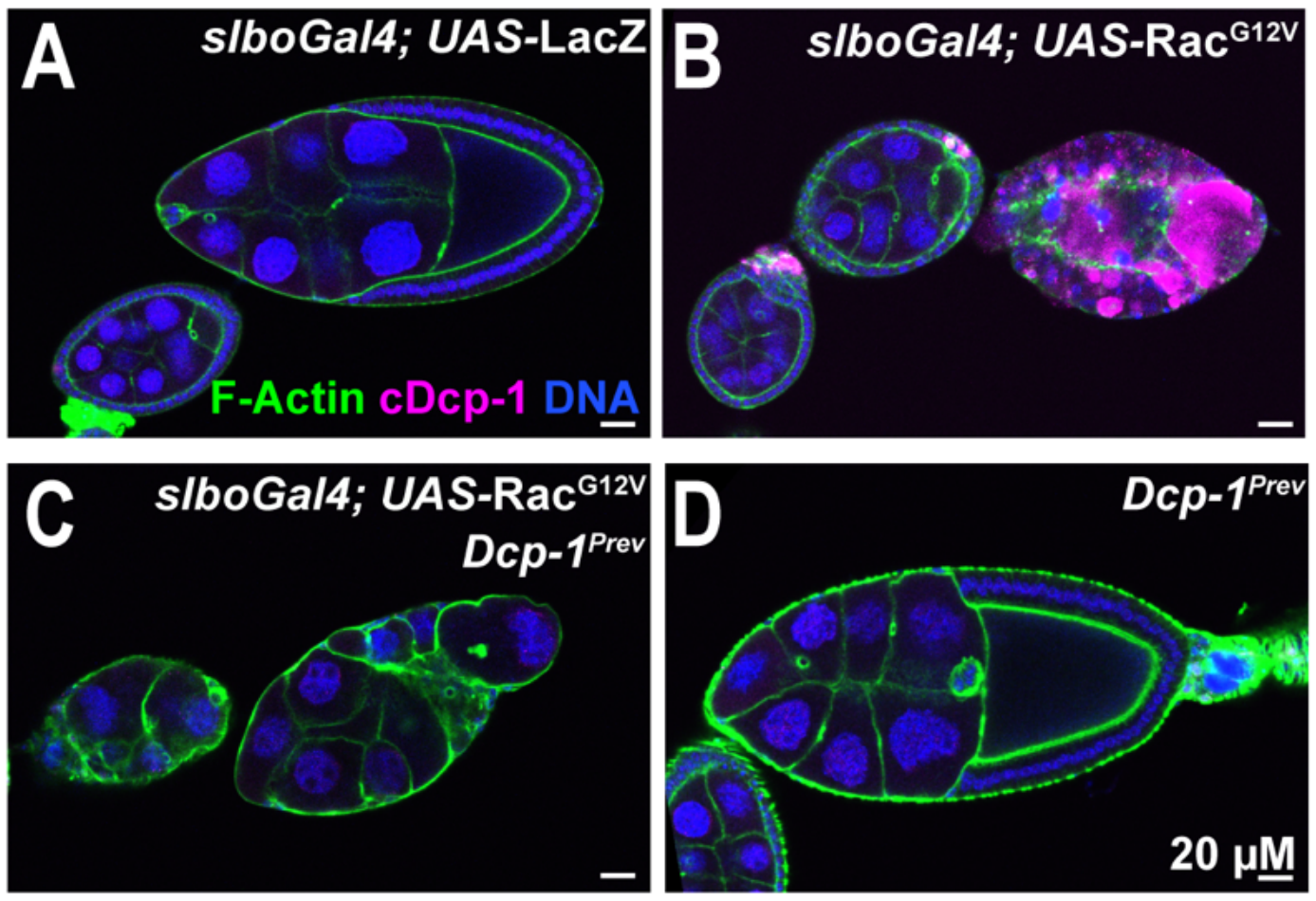

Figure S2.
Suppression of nurse cell chromatin condensation by *Dcp-1*^{Prev}. (A-D) Confocal images of egg chambers from *c306Gal4; Gal80*^{TS} flies stained for cleaved caspase (cDcp-1, magenta), F-actin (green), and DNA (blue) expressing *UAS- LacZ* (A), *UAS-Rac*^{G12V} (B), or *UAS-Rac*^{G12V} in a homozygous *Dcp-1*^{Prev} mutant (C). D shows a homozygous *Dcp-1*^{Prev} mutant egg chamber without hyperactive Rac.

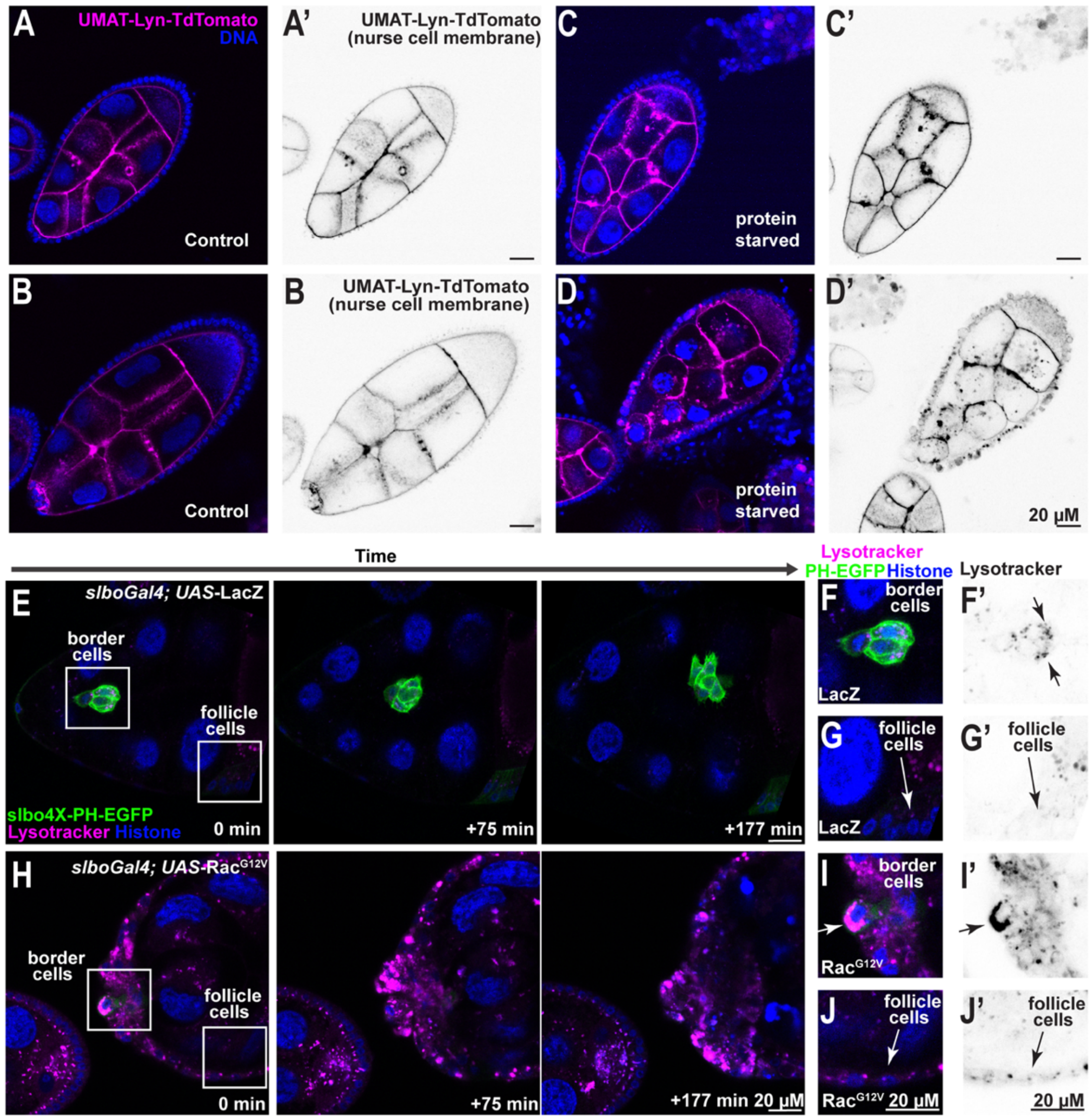

Figure S3.

Nurse cell membrane nibbling and lysotracker accumulation in border cells and other follicle cells prior to germline death. (A-D) Images of fed and protein starved egg chambers stained expressing UMAT-Lyn-TdTomato and stained with Hoechst. (E-F) Images from a timelapse series of *slboGal4;UAS-LacZ (E*) expressing UbHisRFP (blue), *slbo*4x-PH-EGFP (green) and stained for lysotracker-deep red (magenta). (F- F') Magnified image at time=0 minutes highlights lysotracker puncta in the merged image of border cells (F) and inverted fluorescence of lysotracker (F'). (G) Magnified image of follicle cells in *slboGal4;UAS-LacZ* at time=0 mins. (G') Inverted fluorescence of lysotracker. Arrows indicate follicle cells. (H-J) Images from a timelapse series of a *slboGal4;UAS-Rac*^{G12V} (H). (I) Magnified image of Rac^{G12V} border cells at 0 minutes. (I') inverted fluorescence of lysotracker. Arrow indicates accumulation of lysotracker. (J) Magnified image of follicle cells in *slboGal4;UAS-Rac*^{G12V} egg chambers at time=0 mins. (J') inverted fluorescence of lysotracker. Arrows indicate follicle cells. See also Movies S14-15.

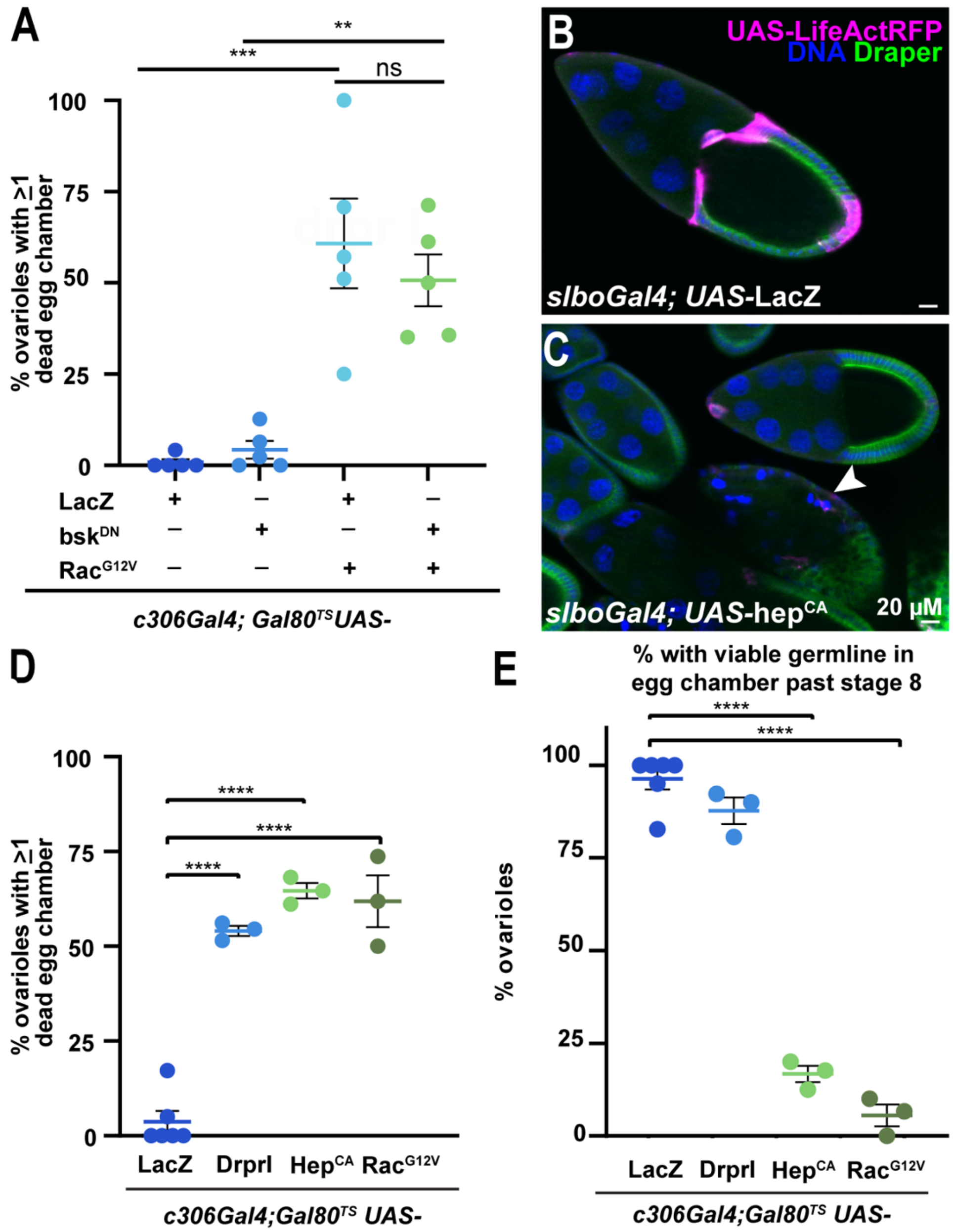

Figure S4.

Expression of Rac^{G12V}, Drpr, or hepCA with border cell driver, *c306Gal4; Gal80^{Ts}* induces germline death (A) Plot showing mean +/- SEM frequencies of percent of ovariole strands with germline death for the indicated conditions. (B-C) Examples of images of ovariole strands for the indicated conditions Scale bar: 20 µm (D) Plot showing mean +/- SEM frequencies of percent of ovariole strands with germline death for the indicated conditions. Plot showing the mean +/- SEM frequencies of ovariole strands with viable egg chambers past stage 8. Each dot represents an experimental replicate analyzing n>18 egg chambers P values are from a One Way ANOVA with Post-Hoc Tukey. ****p<0.0001, **p<0.01, ***p<0.001, ns: not significant.

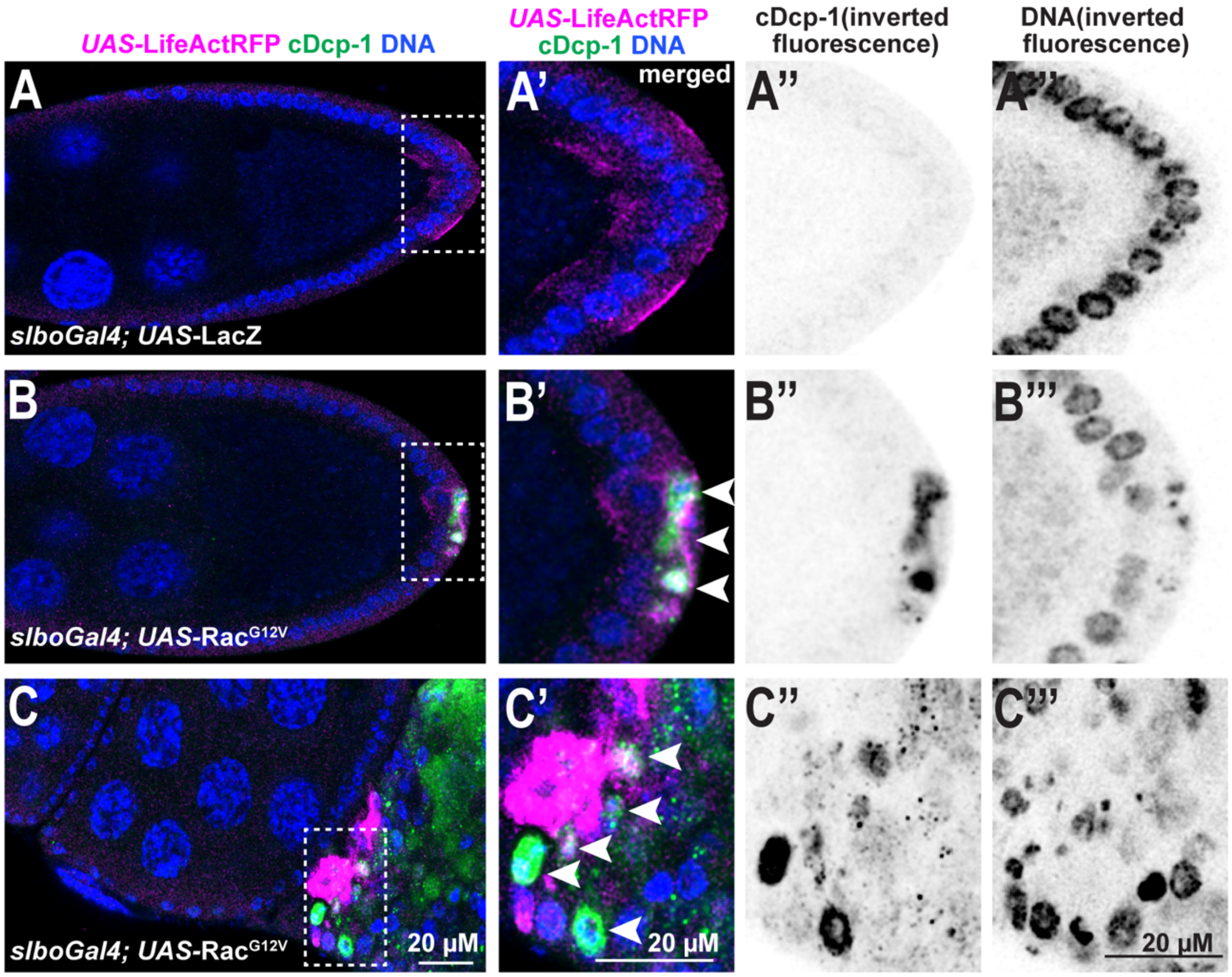

Figure S5.

Follicle cells die and activate cleaved caspase in egg chambers with Rac^{G12V}. (A) Control egg chamber expressing UAS-lacz and LifeActRFP with *slboGaL4*. (A') Magnified image of posterior follicle cells and inverted fluorescence of cDcp-1 (A'') and nuclei stained with Hoechst (A'''). (B) Egg chamber expressing UAS-Rac^{G12V} and UAS-LifeActRFP with *slboGaL4* prior to germline death (B') Magnified image of posterior follicle cells and inverted fluorescence of cDcp-1 (B'') and nuclei stained with Hoechst (B'''). (C) Egg chamber expressing Rac^{G12V} and LifeActRFP with *slboGaL4* that has undergone germline death (C') Magnified image of posterior follicle cells and inverted fluorescence of cDcp-1 (C'') and nuclei stained with Hoechst (C'''). Boxes show magnified areas. Arrowheads point to follicle cells with positive cDcp-1 staining. Scale bars: 20 μm.

Supplemental Movies

Movie S1.

Control border cells accumulate nurse cell membrane puncta. *slboGal4; UAS-LacZ*; *slbo*4XPHEGFP(green, border cell membranes) and UMAT-LynTdTomato (nurse cell membranes, magenta). Single z-slices acquired every 3 minutes. Scale bar: 20 µm.

Movie S2.

Rac^{G12V}-expressing border cells take in larger nurse cell fragments. *slboGal4; UAS-*Rac^{G12V}*; slbo4XPHEGFP* and UMAT-LynTdTomato. Single z-slices acquired every 3 minutes. Scale bar: 20 µm.

Movie S3.

Control border cell migration. Egg chambers expressing *slboGal4; UAS-LacZ, slbo4*XPH-EGFP (green), UMAT-LynTd-Tomato (Cyan), and Ubi-Histone-RFP (magenta). Max z projections of images acquired every 6 minutes. Scale bar: 10 µm.

Movie S4.

Germline death in *slboGal4; UAS-*Rac^{G12V}. Egg chambers also expressed *slbo*4XPH-EGFP (green), UMAT-LynTd-Tomato (Cyan), and Ubi-Histone-RFP (magenta). Max z projections of images acquired every 6 minutes. Scale bar: 10 µm.

Movie S5.

High magnification of control border cell migration in egg chambers from *slboGal4; UAS-LacZ, slbo*4XPH-EGFP (green), UMAT-LynTd-Tomato (Cyan), and Ubi-Histone-RFP flies. Max z projections of images acquired every 6 minutes. Scale bar: 20 µm.

Movie S6.

High magnification of *slboGal4; UAS-Rac*^{G12V}*.* *slbo*4XPH-EGFP (green), UMAT-LynTd-Tomato (Cyan), and Ubi-Histone-RFP (magena), Max z projections of images acquired every 6 minutes. Scale bar: 20 µm.

Movie S7.

Second higher magnification example of *slboGal4; UAS-Rac*^{G12V} *slbo*4XPH-EGFP (green), UMAT-LynTd-Tomato (Cyan), and Ubi-Histone-RFP (magena). Max z projections of images acquired every 6 minutes. Scale bar: 20 µm.

Movie S8.

Control egg chambers retain the nuclear permeability barrier during stage 9. Egg chamber expressing Ubi-GFP.NLS (inverted grayscale fluorescence). Max z projections of images acquired every 5 minutes. Scale bar: 20 µm.

Movie S9.

Posterior germline cell nuclei lose the permeability barrier, and the loss spreads through the syncytium during death. Egg chamber expressing slboGal4; *UAS-Rac*^{G12V} *slbo*4XPH-EGFP Ubi-GFP.NLS (inverted grayscale fluorescence). Max z projections of images acquired every 5 minutes. Scale bar: 20 µm.

Movie S10.

Egg chamber growth during stage 9. Egg chambers expressing, *slboGal4; UAS-LacZ, slbo*4XPH-EGFP (green), UbiHisRFP (magenta). The differential interference contrast (DIC) image is overlaid with fluorescent channels. Single z-slices of images acquired every 5 minutes. Scale bar: 20 µm.

Movie S11.

Egg chamber shrinkage prior to Rac^{G12-} induced chromatin condensation. Time-lapse series from egg chambers expressing slboGal4; *UAS-Rac*^{G12V} *slbo*4XPH-EGFP (green), UbiHisRFP (magenta). The differential interference contrast (DIC) image is overlaid with fluorescent channels.Single z-slices of images acquired every 5 minutes. Scale bar: 20 μm.

Movie S12.

QVD rescues Rac^{G12V} mediated chromatin condensation. Time-lapse series from egg chambers expressing *slboGal4; UAS-Rac*^{G12V} *slbo*4XPH-EGFP (green), UbiHisRFP (magenta) and treated with 100 micromolar QVD for 30 minutes prior to imaging. The differential interference contrast (DIC) image is overlaid with fluorescent channels. Single z-slices of images acquired every 5 minutes. Scale bar: 20 μm.

Movie S13

BAPTA-AM treatment of *slboGal4; UAS-Rac*^{G12V} egg chambers. Time-lapse series from egg chambers expressing *slboGal4; UAS-Rac*^{G12V} *slbo*4XPH-EGFP (green), UbiHisRFP (magenta) treated with 50 micromolar BAPTA-AM. The differential interference contrast (DIC) image is overlaid with fluorescent channels. Single z-slices of images acquired every 5 minutes. Scale bar: 20 μm.

Movie S14.

Lysotracker-positive puncta during normal border cell migration. Timelapse series of a (*slboGal4;UAS-LacZ*) expressing UbHisRFP (blue), *slbo*4PHEGFP (green) and stained for lysotracker-deep red (magenta). Max z-projection of images acquired every 3 minutes. Scale bar: 20 μm.

Movie S15.

Lysotracker accumulation in border and follicle cells during Rac-induced death. Time-lapse series egg chambers expressing *slboGal4;UAS-Rac*^{G12V}, UbHisRFP (blue), and *slbo*4PHEGFP (green) that are stained for lysotracker-deep red (magenta). max z-projection of images acquired every 3 minutes. Scale bar: 20 μm.

Movie S16.

MitoSox staining (magenta) in control egg chambers expressing slboGal4; *UAS-LacZ;UAS-PH(PLCδ)-GFP* (green). Images acquired every 3 minutes.

Movie S17.

MitoSox staining (magenta) in *slboGal4;UAS-;Rac*^{G12V}*;UAS-PH(PLCδ)-GFP* (green) egg chambers. Images acquired every 3 minutes.

Materials and Methods

Fly husbandry and genetics

Flies were maintained in cornmeal-yeast food. Crosses were kept at 18 °C for stocks with *Gal80*^{TS} while other crosses were kept at room temperature or 25 °C and transferred to fresh food every 2-3 days. Control and experimental conditions were kept at the same temperature for each experiment. Prior to ovary dissections, 5-7 female flies and 1-2 males were incubated together with supplemental yeast at 29 °C overnight. For protein starvation, flies were kept on vials with apple juice agar at 29 °C overnight. For FlpOUT experiments, flies were heat-shocked at 37°C for 30 minutes and fattened overnight at 29°C.

Live imaging

Whole ovaries were first dissected with forceps from flies in live imaging media [Schneider's media (Thermo Fisher Scientific) supplemented with 20% heat-inactivated Fetal Bovine Serum (Sigma-Aldrich) and 1x antimycotic/antibiotic (VWR) at pH 6.85-6.95] in a depression slide as described (64). Then, individual ovarioles were teased from the muscle sheath by holding the germarium using forceps. Dissected ovariole strands were resuspended in live imaging media with 0.4 mg/mL bovine insulin (I1882, Sigma-Aldrich) and 0.1 % agarose. For lysotracker and dextran staining, ovaries were incubated at 1:1000 immediately prior to live imaging. For caspase inhibition, ovarioles were incubated in 100 µM Q-VD-OPh (APExBio) in live imaging media for 30 minutes on a rocker prior to imaging. BAPTA-AM, cell permeant Ca^{2+} chelator (ThermoFisher) was incubated for 45 minutes on a rocker prior to live imaging at a concentration of 50 micromolar 0.02% with Pluronic F-127 (Invitrogen). MitoSOX Red (Thermofisher) was added at a concentration of 5uM and incubated for 10 mins with resuspension every 5 minutes. Xrhod-1(Invitrogen) was added at a concentration of 5 micromolar. These dyes and inhibitors were maintained in the culture media throughout imaging. A 20x20 mm coverslip (No. 1.5 Fisherbrand) was split in half to provide space between the imaging coverslip and the sample, and 4 uL of live imaging media was used to stabilize each part of the coverslip 100 microliters of media with the egg chambers was loaded into the center of the split 20x 20 cover slips on a Lumox dish (Sarstedt). A larger cover slip (40X20 mm No. 1.5 Fisherbrand) was then placed over the sample. The sample was inverted to allow

samples to settle close to the coverslip. After 5 minutes, 20 uL of halocarbon oil was added around the periphery of the coverslip.

Samples were imaged on a ZEISS LSM 800 or ZEISS 980. Images shown in Figure 3A-E were acquired on a ZEISS LSM 780 in Lambda mode and images were processed by spectral unmixing to distinguish fluorescent markers with overlapping emissions spectra.

Immunostaining

Ovarioles were dissected from ovaries in live imaging media, allowed to settle, and fixed in 4% paraformaldehyde in 1X PBS for 15 minutes at room temperature. Ovarioles were washed 3 times with 1x PBST at room temperature, and then incubated in primary antibodies overnight (Drpr (DSHB, 1:50), DCAD2, (DSHB, 1:15), pJNK (Cell Signaling Technologies 1:200), cDcp-1 (Cell Signaling Technologies, 1:200) at 4°C. The next day, ovarioles were washed thrice with 1 X PBST and incubated with secondary antibodies at 1:200 (Goat anti-Rat IgG [H+L] Cross-Adsorbed Secondary Antibody, Alexa Fluor 647, 488, or 568, Invitrogen, Goat anti-Mouse IgG [H+L] Cross-Adsorbed Secondary Antibody, Alexa Fluor 488 and 568; Invitrogen) at room temperature. Samples were also incubated with phalloidin-Atto-647N (1:500; Sigma-Aldrich), and Hoechst (1:1000 of 10 mg/mL stock, Invitrogen). Samples were incubated with secondary antibodies, phalloidin and Hoechst together for 1 hour at room temperature. Ovaries were washed with 1X PBST three times then suspended in Vectashield (H-1000; Vector Laboratories) and incubated overnight at 4°C before mounting onto slides.

Image analysis

Egg chamber death scoring

In fixed imaging, death was scored per ovariole, i.e. if there was one or more dead egg chamber(s) at the end of an ovariole strand. An egg chamber was scored as dead when the germline chromatin (DNA labeled with Hoescht) became condensed and/or fragmented. In live samples of stage 9 egg chambers, death was scored when the germline chromatin, labeled with histone RFP, underwent condensation and/or fragmentation prior to the end of the imaging session (within 4.5 hours for Figure 4 and within 5.5

hours for Figure 3). Chromatin condensation was defined at when histone signal became enriched in spots within the nuclear boundaries while chromatin fragmentation occurred when the chromatin condensates begin to split apart and spread from the nucleus.

Nurse cell membrane analysis

Nurse cell membrane area was analyzed from a single z-stack or live egg chambers. The area of nurse cell membrane inside the border cells was measured by drawing an ROI around the nurse cell membrane fragment in the border cells, using *slbo*4X-PH-EGFP or follicle cells in Image J. The largest membrane fragment is shown for each egg chamber.

Germline area measurements

The entire area of the nurse cells were traced in Image J using the DIC images from movies with UbiHis-RFP and *slbo*4XPH-EGFP every 25 minutes until the germline died (indicated by chromatin condensation) or moved excessively or of the field of view. The average change in area per 25 minutes is reported for each egg chamber.

Migration Index

To measure migration indices, the location of the border cell cluster was assessed in stage 10 egg chambers, defined as when the oocyte occupies ½ of the egg chamber volume and the anterior border of the columnar follicle cells aligns with the anterior oocyte border. The position of the border cell cluster was manually scored in the following categories: 0%- still attached at the anterior end of the egg chamber; <50% - detached but located in the anterior half of the migration path; >50% - within the posterior half of the migration path; and 100% - at the oocyte border.

Fluorescence reporters and intensity assays

To measure the fluorescence intensity of Drpr border cells at different stages, a small box (10 x 10 pixels) was drawn around the border cell cluster, and an equivalent box was selected outside of the egg chamber for background subtraction, for each image. For pJNK, a 20 x 20 pixel box was drawn

around a GFP+ cell and a GFP- cell within the same egg chamber, and an equivalent box was selected outside of the egg chamber for background subtraction. The ratio was calculated by the (mean fluorescence intensity GFP+ cell - background)/(mean fluorescence intensity of the GFP- cell).

The percentage of cDcp-1 positive and *puc*-LacZ positive cells scoring were scored manually. cDcp-1 was considered positive if it accumulated within the germline (usually observed at the periphery of the nurse cells, which is consistent with prior reports using this staining. *puc*-LacZ was considered positive if there was a detectable enrichment of the signal within the nucleus. Border cells (bcs) or posterior follicle cells (pfcs) were scored as *puc*-LacZ positive if there was one or more in an egg chamber.

Data representation and statistical analysis

All sample sizes and middle bars are reported in the figure legends. For analysis the inclusion criteria were all undamaged samples, correct egg chamber stage, and in-focus images. Exclusion criteria include damaged, out of focus, or incorrect stage time points and images. For live imaging, all stage 9 egg chambers were included unless they were damaged by dissection, went out of view or focus due to movement during the imaging or if they became abnormally flattened during the imaging session. Statistical tests used are reported in the figure legends * indicates $p<0.05$, ** $p<0.01$. *** $p<0.001$, **** $p<0.0001$. Image processing differences are noted in figures and legends but were generally processed the same way as controls.
